# Defining Keypoints to Align H&E Images and Xenium DAPI-Stained Images Automatically

**DOI:** 10.3390/cells14131000

**Published:** 2025-06-30

**Authors:** Yu Lin, Yan Wang, Juexin Wang, Mauminah Raina, Ricardo Melo Ferreira, Michael T. Eadon, Yanchun Liang, Dong Xu

**Affiliations:** 1 School of Artificial Intelligence, Jilin University, Changchun 130012, China; 2 Department of Electrical Engineering and Computer Science, Christopher S. Bond Life Sciences Center, University of Missouri, Columbia, MO 65211, USA; 3 Key Laboratory of Symbol Computation and Knowledge Engineering of Ministry of Education, College of Computer Science and Technology, Jilin University, Changchun 130012, China; 4 Department of Biomedical Engineering and Informatics, Indiana University Indianapolis, Indianapolis, IN 46202, USA; 5 Department of Medicine, Indiana University Indianapolis, Indianapolis, IN 46202, USA; 6 School of Computer Science, Zhuhai College of Science and Technology, Zhuhai 519041, China

**Keywords:** spatial transcriptomics, Xenium technology, H&E image, image alignment, nucleus segmentation, graph matching

## Abstract

10X Xenium is an in situ spatial transcriptomics platform that enables single-cell and subcellular-level gene expression analysis. In Xenium data analysis, defining matched keypoints to align H&E and spatial transcriptomic images is critical for cross-referencing sequencing and histology. Currently, it is labor-intensive for domain experts to manually place keypoints to perform image registration in the Xenium Explorer software. We present Xenium-Align, a keypoint identification method that automatically generates keypoint files for image registration in Xenium Explorer. We validated our proposed method on 14 human kidney samples and one human skin Xenium sample representing healthy and diseased states, with expert manually marked results. These results show that Xenium-Align could generate accurate keypoints for automatically implementing image alignment in the Xenium Explorer software for spatial transcriptomics studies. Our future research aims to optimize the method’s runtime efficiency and usability for image alignment applications.

## 1. Introduction

Spatially resolved transcriptomics (SRT) technology has rapidly developed and been widely applied in recent years [1,2,3]. These high-resolution technologies, including 10X Genomics Xenium, NanoString CosMx Spatial Molecular Imaging (SMI), and many more [4,5,6,7], offer spatial information at single-cell and subcellular resolution, providing effective approaches to study tissue architecture and biological functions [8,9]. The Xenium Explorer software, maintained by 10X Genomics, is widely used to visualize the nucleus and cell boundary details for Xenium sequencing samples [10,11,12,13]. To validate the sequencing quality, researchers usually need to manually align Xenium DAPI-stained images and Hematoxylin & Eosin (H&E) images, which usually takes extensive time and labor. The alignment in Xenium Explorer requires precise manual placement or an imported file of matched keypoints, which serve as anchors for matching all the cells. The prepared keypoint file helps enable automatic image alignment in Xenium Explorer.

Matched keypoints are identified by recognizing shared cell nuclei between the DAPI-stained and H&E images. The positions and boundary coordinates of the cell nuclei can be directly obtained from pre-processing the DAPI-stained image. In the imported H&E image, the location information of cell nuclei is usually derived from image segmentation [14,15,16]. Several image segmentation models have been successfully proposed. Cellpose [17], a generalist deep learning model, can segment a wide range of cell types without requiring model retraining or parameter adjustments. StarDist [18] applies a light-weight neural network of U-Net [19] and the shape representation based on star-convex polygons to localize the cell nuclei. DeepCell Kiosk [20] uses a cloud-based method to dynamically scale deep learning workflows for large imaging datasets. Squidpy [21] is a Python-based framework that can enable community-driven, scalable analyses for spatial transcriptomics data. These effective methods of image segmentation offer promising approaches for precisely localizing the cell nuclei in H&E images.

To align the DAPI-stained image and H&E image, the same location points must serve as anchors between the two images for image registration. Cropping image patches for each nucleus and evaluating their similarities among different patch pairs is an applicable approach. Several widely used image quality assessment measures in digital image processing systems [22,23,24], including the peak signal-to-noise ratio (PSNR) [25], structure similarity index measure (SSIM) [26], and mean square error (MSE) [27], can be used to measure the reconstruction quality and similarity degrees between a DAPI-stained image and an H&E image. To filter out the outliers for the initially matched point pairs, the Delaunay triangulation graph and nucleus polygon matching methods are applied. Delaunay triangulation graph matching aims to evaluate the topological consistency between two graphs. It checks whether the connected edges are identical for each node based on their index order. Nodes with inconsistent edge connections are removed, and the graphs are subsequently updated. The matching method iteratively performs this process and terminates when all nodes achieve identical edge connectivity. Nuclei polygon matching primarily evaluates consistency in the shape and size between two cell nuclei. Cell nuclei with insufficient overlap ratios are deleted during the matching process.

Xenium Explorer is a practical and widely used visualization and analysis software. Compared to other tools, such as Fiji, it demonstrates superior advantages. Fiji is an open-source biomedical image analysis platform that focuses on processing and visualizing scientific images in bioinformatics. Xenium Explorer is specially designed for Xenium data used in various spatial transcriptomics analyses. The functions of integrating images and transcripts are built into the software, such as cell typing, gene expression visualization, and so on. However, to achieve these functions, extra plugins or extensions are required in Fiji.

At present, users have to spend significant time and effort to identify the matched keypoints during the image alignment process in Xenium Explorer software. No effective method has been proposed to address the labor-intensive issue of manually placing keypoints in the software for precise image alignment. To enable accurate and automatic keypoint identification and facilitate the alignment between the H&E image and Xenium DAPI-stained image for more efficient spatial transcriptome analyses, this study presents a keypoint identification method, named Xenium-Align, to generate the keypoint file for automatic image alignment in Xenium Explorer software. The proposed method includes the function modules of image processing, multi-directional enhanced image assessment, Delaunay triangulation graph matching, nucleus polygon matching, and keypoint file generation. The image segmentation model is used to localize cell nuclei in the H&E image, which is imported and aligned to the Xenium DAPI-stained image in Xenium Explorer. The cropped image patches are used to describe cell nuclei regions in both segmented H&E images and extracted DAPI-stained images. We apply the image quality assessment index of PSNR to search for the matched locations of cell nuclei. Two matching methods, Delaunay triangulation graph matching and nucleus polygon matching, are used to remove the outliers. We finally output the accurate keypoint file in a CSV format, which can be imported into Xenium Explorer software. Xenium Explorer requires at least three keypoints in the imported file and applies an algorithm [28,29] based on the least-squares solution to implement image alignment. The more accurate the keypoints, the better the image alignment performance.

Fourteen human kidney samples and one human skin Xenium sample were used to validate our proposed keypoint identification method. According to the experimental results, Xenium-Align can precisely identify the matched cell nuclei between the DAPI-stained image and H&E image to generate a keypoint file for automatically implementing image alignment in Xenium Explorer software. The results also further demonstrate the usability of Xenium Explorer software and the superiority of our proposed method in reducing labor costs for spatial transcriptome analyses.

## 2. Materials and Methods

**Figure 1:**
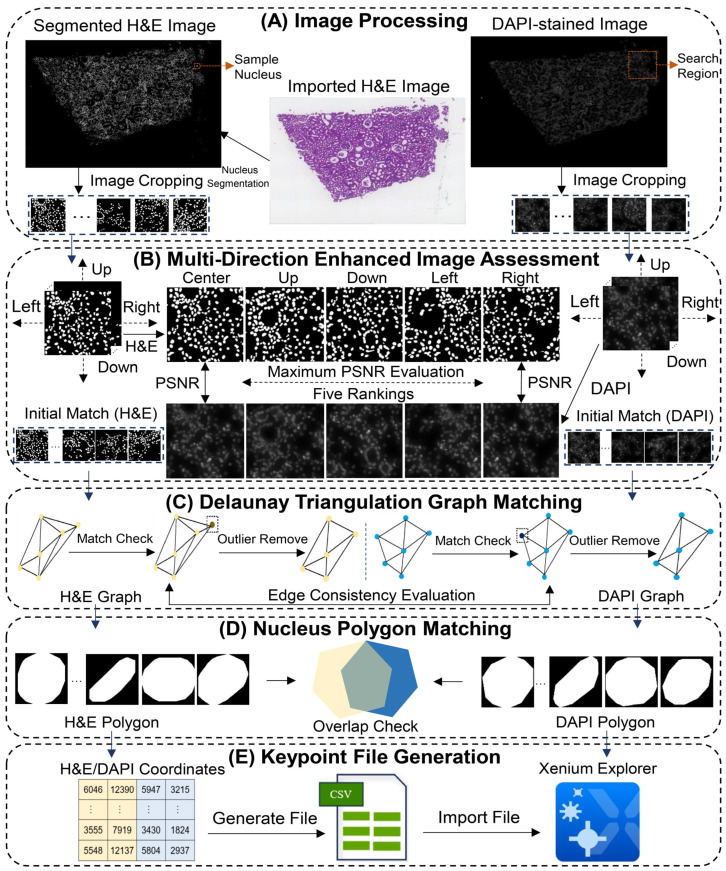
The workflow of our proposed keypoint identification method, named Xenium-Align, automatically generates the keypoint file for the Xenium Explorer software. The image alignment details of the five experimental parts are as follows: (**A**) shows the image processing on the imported H&E image and the extracted DAPI-stained image. Nucleus patches are cropped with the same radius size from the segmented H&E image (by Cellpose or StarDist models) and the extracted DAPI-stained image to locate nucleus positions. (**B**) Details of the multi-directional enhanced image assessment involving five aspects (center, up, down, left, and right moving directions). The image quality evaluation index of PSNR is used to measure the similarity between the H&E patch and the DAPI-stained patch. (**C**) Shows the Delaunay triangulation graph matching process used to filter out the outliers due to the mistaken placement of the initially matched keypoints. (**D**) Shows nucleus polygon matching by evaluating the overlap degree between the cell nuclei from the DAPI-stained and H&E images to further remove mismatched keypoints. (**E**) Illustrates the output of placement points with spatial coordinates kept in a CSV format file, which can be used as the input for image alignment in Xenium Explorer software. This figure was prepared using Adobe Illustrator CC Software (version 2017; Adobe Inc., San Jose, CA, USA).

The main workflow of Xenium-Align is designed to generate a keypoint file for image alignment between the Xenium DAPI-stained image and the H&E image in Xenium Explorer software. Figure 1 illustrates the framework of our proposed keypoint identification method to automatically derive the coordinates of keypoint placements. The overall workflow of Xenium-Align includes the following five steps: (A) Image processing on the imported H&E image and the extracted DAPI-stained image to crop patches based on the nucleus center point with the same radius sizes. Cellpose or StarDist is used as the optional nucleus segmentation model for the H&E image. (B) Multiple moving positions (center, up, down, left, and right) on the cropped patches are used to enhance image quality evaluation based on the PSNR assessment index. After ranking and performing an enhanced evaluation, the initially matched patches of cell nuclei can be obtained. (C) Building a Delaunay triangulation and implementing graph matching with a topology consistency evaluation to remove the outlier cell nuclei. (D) Locating each nucleus pair and measuring the polygon overlapping degrees to keep the matched pairs that come from the same cell. (E) Generating the coordinates of keypoint placements in the H&E image and the DAPI-stained image as a CSV format file. The keypoint file can finally be imported manually into the Xenium Explorer software (version 3.2.0).

### 2.1. Image Processing

For the imported H&E image in Xenium Explorer, we first rotate the image direction by evaluating the assessment index of the MSE between the DAPI-stained image (in black-and-white) and the H&E image to ensure consistency with the image layout. Xenium-Align applies a nucleus segmentation model to perform image segmentation in H&E images for localizing the cell nuclei. The nucleus segmentation image can be obtained using Cellpose or StarDist models on each sample. In the segmentation image, a segment includes some labels and represents one nucleus, which is applied to derive the coordinate values of each nucleus. For the DAPI-stained image in Xenium Explorer, we extract the gray-scale image from the cell morphology data stored as the OME-TIFF format by the tifffile Python package [30]. The polygon vertices of the cell nuclei boundaries used to describe each nucleus can be obtained using the spatialdata-io Python package (version 0.1.4) [31]. The mean of the maximum and minimum coordinate values in width and length is used as the center point for each nucleus. We crop the same radius size based on the coordinates of the center points to represent the cell nuclei in both the H&E image and the DAPI-stained image.

To search for the matched cell nuclei from the H&E image to the DAPI-stained image, we define a search region in the DAPI-stained image for each nucleus in the H&E image. The coordinates of the center points of each search region are based on the proportions of the x-coordinate and y-coordinate to the width and length in the H&E image [32]. The x-coordinate of the center points in the search region is defined as follows:

(1) $$X_{DAPI}=W_{DAPI}\frac{X_{H\&E}}{W_{H\&E}}$$

where *W* and *X* denote the width and x-coordinate of the corresponding image, respectively. The y-coordinate of the center points in the search region is defined as follows:

(2) $$Y_{DAPI}=L_{DAPI}\frac{Y_{H\&E}}{L_{H\&E}}$$

where *L* and *Y* denote the length and y-coordinate of the corresponding image, respectively. After setting a search radius, a square search region that includes a number of cell nuclei can be obtained from a DAPI-stained image.

### 2.2. Multi-Directional Enhanced Image Assessment

We apply the PSNR index, which is a valid image quality measurement, to evaluate the matching degree among the cropped patches from the imported H&E image and the Xenium DAPI-stained image. The PSNR index can reliably quantify the reconstruction quality and calculate image similarity [33]. It is defined via MSE, which is another well-known objective index measuring the quality change between images. Given gray-scale images *a* and *b*, their image sizes are both *M* × *N*. MSE is defined as follows:

(3) $$MSE(a,b)=\frac{1}{MN}\sum_{i=1}^{M}\sum_{j=1}^{N}{\left(a_{ij}-b_{ij}\right)}^{2}$$

where *a_ij_* and *b_ij_* denote the pixel values in each position of the two images. The smaller the MSE value, the higher the image quality. The PSNR index between *a* and *b* is defined as follows:

(4) $$PSNR(a,b)=10\log_{10}(\frac{L^{2}}{MSE(a,b)})$$

where *L* is the maximum pixel value of the image. A larger PSNR value indicates more similarity between the two assessed images.

As shown in Figure 1B, four more patches from four directions of the cropped nucleus patch in the segmented H&E image can be obtained by moving the x-coordinates or y-coordinates in four directions (up, down, left, and right), based on the center points. For each nucleus patch in the H&E image, we evaluate the PSNR indexes between it and the cropped nucleus patches in the search region of the DAPI-stained image from five aspects (different moving directions). The five moving directions increase the range of the image signal and the region for each nucleus patch, which helps define accurate matches. After obtaining five rankings of PSNR indexes for each nucleus in the H&E image, the cell nuclei with the maximum PSNR values are considered matched candidates in each moving direction, which is defined as follows:

(5) $$PSNR_{nucleus}=\max(PSNR_{1},PSNR_{2},\ldots ,PSNR_{k})$$

where *k* denotes the number of cell nuclei in the search region for a certain moving position. *PSNR_nucleus_* denotes the maximum value in the current ranking. If the cell nuclei with maximum PSNR values in the five rankings of different moving directions are the same, the cell nuclei in the DAPI-stained image and H&E image are kept as initially matched pairs. The center points of these retained cell nuclei, with the same quantity in the H&E image and the DAPI-stained image, are considered as initially matched keypoints.

### 2.3. Delaunay Triangulation Graph Matching

The Delaunay triangulation is a critical computational method that can represent the geometrical and topological relationships of the spatial points that are connected [34,35,36]. It can be effectively used in many applications for capturing network structures [37,38,39] and conducting matching methods [40,41]. In this study, we remove the keypoint outliers via the matching method based on the Delaunay triangulation graph.

As shown in Figure 1C, after obtaining the initially matched keypoints, we build two Delaunay triangulation graphs using the center coordinates of initially matched cell nuclei, referencing the construction method [42] for the H&E image and DAPI-stained image, respectively. Given two Delaunay triangulation graphs, $G_{H\&E}=(V_{H\&E},E_{H\&E})$ and $G_{DAPI}=(V_{DAPI},E_{DAPI})$, VH&E and VDAPI are built; *V_H&E_* and *V_DAPI_* are the sets of nodes in the graphs, and *E_H&E_* and *E_DAPI_* are the sets of edges in the graphs, respectively.$(v_{i}^{H\&E},v_{j}^{H\&E})\in E_{H\&E}$ and $(v_{i}^{DAPI},v_{j}^{DAPI})\in E_{DAPI}$ capture the edges between node *i* and node *j* in the graphs of the two images. Figure S1 details the matching process of evaluating the edge consistency between two graphs to remove the mismatched nodes and make the same nodes and edges.

As shown in Figure S1A, two edge sets can be obtained for the graphs of the H&E image and the DAPI-stained image. The initially matched cell nuclei are indexed as nodes in the graph. The edges in two sets are checked by evaluating the node consistency according to the order of node indexes. When inconsistent edges are found, the node with the smaller first index in the two evaluated edges is removed; for example, the node with index 0 in the two graphs is deleted during the matching process. After several matching evaluations, as shown in Figure S1B, the two matched edge sets with consistent nodes and edges between the two graphs can be generated. The kept nodes in two graphs are used as the final output for the next matching method, nucleus polygon matching. Delaunay triangulation graph matching can ensure the consistency of the overall topology relationship and mutual distances between cell nuclei in the two graphs of the H&E image and the DAPI-stained image.

### 2.4. Nucleus Polygon Matching

The concept of a polygon can be used to represent cell nuclei based on the boundary points from nucleus segmentation models [18,43,44], where the nucleus polygon reflects the shape features of cell nuclei. Matching the nucleus polygon can be a further measure of whether two nuclei come from the same cell or not. In this study, the nucleus polygon matching method is used to further filter out keypoint outliers from the output of Delaunay triangulation graph matching.

As shown in Figure 1D, after obtaining output pairs with the keypoints of initially matched cell nuclei from Delaunay triangulation graph matching, we apply nucleus polygon matching to further filter out the incorrectly matched cell nuclei in the keypoints. For the cell nuclei of keypoints in the H&E image, the segment labels of the nucleus segmentation model are used to derive the smallest convex sets that contain all the points for each nucleus using the Quickhull algorithm [45] to construct the polygons. For the cell nuclei of keypoints in the DAPI-stained image, the coordinates of convex sets can be obtained from the original Xenium data. The cell nuclei polygons from the H&E image and the DAPI-stained image are mapped into the coordinate space with the same origin points.

Next, the overlap degree based on the areas of intersection between two matched cell nuclei polygons is calculated. As we have the cell nuclei polygons of the H&E image and the DAPI-stained image, the intersection area is defined as follows:

(6) $$Area_{intersection}=S_{H\&E}\cap T_{DAPI}$$

where *S* and *T* denote the overall polygon areas of the H&E image and the DAPI-stained image, respectively. We use the average or minimum proportion of the intersection area to the overall polygon areas of *S_H&E_* and *T_DAPI_* as a measurement index to evaluate the overlap degree between two cell nuclei polygons from the H&E image and the DAPI-stained image. The overlap index based on the average proportion is calculated as follows:

(7) $$I_{overlap}=\frac{1}{2}(\frac{Area_{intersection}}{S_{H\&E}}+\frac{Area_{intersection}}{T_{DAPI}})$$

And the overlap index based on the minimum proportion is calculated as follows:

(8) $$I_{overlap}=\min(\frac{Area_{intersection}}{S_{H\&E}},\frac{Area_{intersection}}{T_{DAPI}})$$

As for both overlap indexes, the larger the overlap index value, the greater the similarity in shape features between the two polygons. It further indicates the higher probability that two nuclei come from the same cell. Compared with the polygon matching condition based on the average overlap index, the condition using the minimum overlap index is improved, because it requires that the intersection area of the two polygons meet the specified minimum threshold.

### 2.5. Keypoint File Generation

A transformation matrix file and a keypoint alignment file in CSV format are needed for the Xenium Explorer software for image alignment. In this study, we focus on generating the keypoint alignment file for automatic image alignment.

When we obtain the final keypoints of the matched cell nuclei, processed through image processing, multi-directional enhanced image assessment, Delaunay triangulation graph matching, and nucleus polygon matching, the center points of the cell nuclei in the H&E image and the DAPI-stained image are used as the spatial coordinates to be saved in the keypoint alignment file. The row-by-row formatting introduced in the official tutorial [29] of image alignment is applied to save the coordinate values of matched pairs of the cell nuclei between the Xenium DAPI-stained image and the aligned post-Xenium image in the keypoint alignment file. The final generated keypoints are saved in a CSV file and can be manually imported to Xenium Explorer under the computer mouse option ‘Yes, use an existing alignment file’, which reduces labor costs. Figure S2 shows the saved format of a keypoint file that can be imported into Xenium Explorer for implementing image alignment. As shown in Figure S2, the Fixed and Alignment columns are the coordinate values of keypoints in the DAPI-stained image and the H&E image, respectively.

### 2.6. Datasets and Experimental Settings

In this study, we used two Xenium datasets generated from human kidney and human skin tissues to validate our proposed identification method. One dataset included 12 Xenium samples; these samples were divided into three conditions, as follows: healthy kidney samples (REF), samples with chronic kidney disease (CKD), and samples with the autoimmune disease, lupus (SLE). The 12 human kidney samples used the fresh frozen (FF) [46,47] method to preserve the tissue section and were labeled from F59 to 5582. Supplementary Table S1 shows more details about this Xenium dataset. To further test the effectiveness of our proposed identification method, the other Xenium dataset that included two human kidneys and one human skin sample with the formalin-fixed, paraffin-embedded (FFPE) [48,49,50] preservation method was used as the test dataset. There were 56,510, 97,560, and 90,106 cells in these three samples (kidney cancer, non-diseased kidney, and non-diseased skin cases, respectively). The proposed identification method, Xenium-Align, was implemented in Python 3.9.2. All operating programs were conducted on a computing server running the Ubuntu 18.04 operating system, with a 2.2 GHz, 144-core CPU, 503 GB RAM, and an NVIDIA TU102 [TITAN RTX] GPU. The Xenium Explorer software used to visualize the performance of image alignment is version 3.2.0.

Tables S2 and S3 detail the hyperparameter settings and the segmentation results of the nucleus segmentation models for the 12 human kidney samples using the FF preservation method, respectively. Cellpose was used as the primary method to implement nucleus segmentation for tracking the locations of cell nuclei in the imported H&E images. As for the hyperparameter settings in the Cellpose model, we used channel 1 of the H&E image to segment cell nuclei. The hyperparameter defines the minimum number of pixels per mask as 15, and the hyperparameter that controls whether all cells with errors below the threshold are kept is set to 0.8. In the case of ineffective segmentation results by the Cellpose model, where the segment ratio is lower than 50 percent, the StarDist model is used, and the hyperparameter in StarDist that performs non-maximum suppression for polygons above the object probability threshold is set to 0.3.

We sample 100 cells from the segmented cell nuclei of the H&E image in each epoch to implement Xenium-Align. We set the minimum number of keypoints that our proposed identification method finally outputs as 15. When setting the search region in the DAPI-stained image, the region radii are set to ratios of 0.06, 0.125, and 0.5, relative to the minimum value in the width and length of the DAPI-stained image, respectively. When we obtain a search region, the minimum number of cells in the region is set to 50 to evaluate whether to keep it or not. The radius size used to crop patches for cell nuclei is 400 pixels, and the move distance based on the center coordinates in the four directions (up, down, left, and right) is 300 pixels. All the cropped patches are resized to a resolution of 224 × 224 for the processing of multi-directional enhanced image assessment. For nucleus polygon matching, we set the average overlap threshold value to 0.9 to evaluate whether two nuclei came from the same cell. The details about the hyperparameter settings for each Xenium sample using the FF preservation method are shown in Table 1.

To further improve the nucleus polygon matching, the minimum overlap index was used, and the threshold value was set to 0.92 to implement Xenium-Align. For the two human kidney samples and one human skin sample using the FFPE preservation method, Cellpose was used to conduct nucleus segmentation, and all hyperparameter settings were kept the same as those used in the 12 samples with the FF method, except that the size of the radius for cropping patches for cell nuclei was set to 200 pixels, the move distance in the four directions was set to 150 pixels, and the minimum number of generated keypoints was set to 10.

## 3. Results

Our proposed keypoint identification method, Xenium-Align, enables precise keypoint identification to generate a keypoint file for automatic image alignment in Xenium Explorer software (version 3.2.0). The experimental results demonstrate that Xenium-Align precisely locates the matched cell nuclei and generates a keypoint file through the functional modules of image processing, multi-directional enhanced image assessment, the Delaunay triangulation graph, nucleus polygon matching, and keypoint file generation.

### 3.1. Image Alignment by Xenium-Align in Xenium Explorer Software

**Figure 2:**
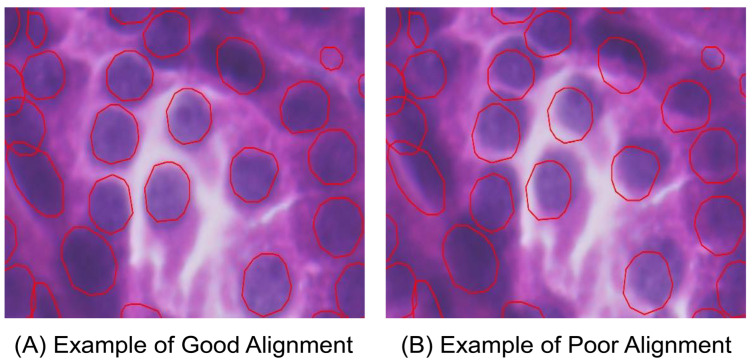
Visualization results of image alignment in good and poor cases using our proposed keypoint identification method on the same tissue region on sample F59. (**A**) Shows an example of good image alignment; (**B**) shows an example of poor image alignment.

**Figure 3:**
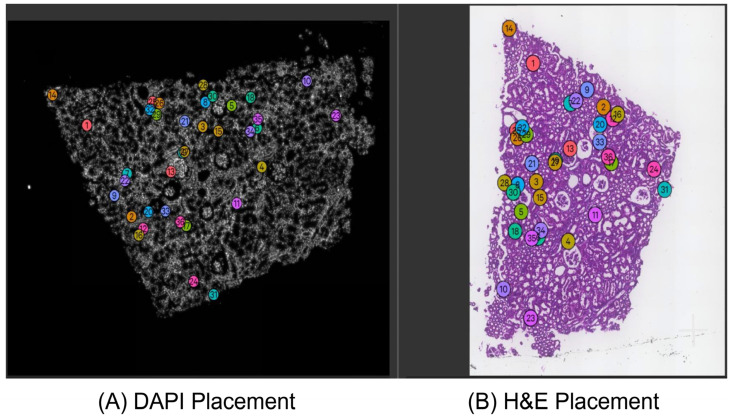
Visualization results of the keypoint placements across the whole tissue between the H&E image and the DAPI-stained image using our proposed identification method in Xenium Explorer, from sample F59. (**A**) Shows the keyponit placement of the DAPI-stained image; (**B**) shows the keypoint placement of the H&E image.

**Figure 4:**
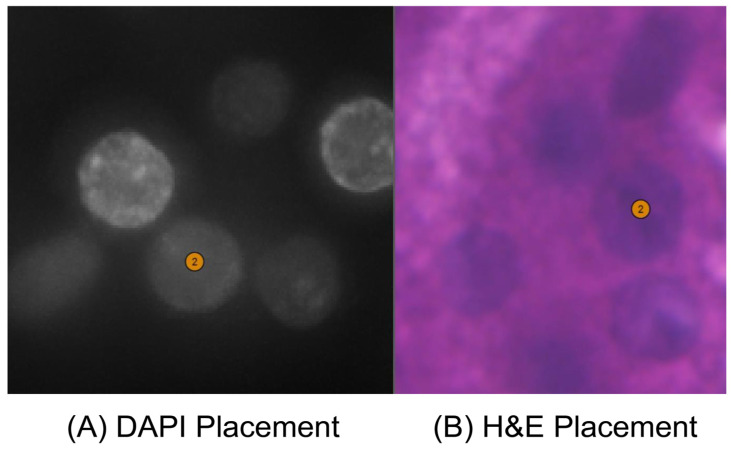
Visualization results of the accurate keypoint placements of a certain pair of keypoints between the H&E image and the DAPI-stained image using our proposed identification method in Xenium Explorer, from sample F59. (**A**) Shows the keypoint placement of the DAPI-stained image; (**B**) shows the keypoint placement of the H&E image. The number “2” in the figure indicates the corresponding spots in Figure 3.

Table 1 shows the details of the hyperparameter settings for our proposed keypoint identification method and the results of keypoint generation on 12 human kidney samples using the FF preservation method. We randomly set the number of false keypoints to evaluate the threshold number that obviously influences the image alignment performance. The number of false keypoints was tested from ten percent of the total number of keypoints to the total keypoint number in intervals of ten percent. The specific tested percentage values of the number of false keypoints to the total keypoint number are 10%, 20%, 30%, and so on. Table 2 details the results of a specific number of accurate keypoints and the threshold number of false keypoints, respectively. Figure 2 illustrates the visualization performances of good and poor image alignments in Xenium Explorer software from the same tissue region on sample F59. The poor image alignment is based on the keypoint file, including the threshold number of false keypoints. Figure 3 shows the keypoint placements across the whole tissue between the H&E image and the DAPI-stained image using our proposed identification method in Xenium Explorer software on sample F59. Figure 4 and Figure S3, respectively, show the accurate and false placements of certain pairs of keypoints between the H&E image and the DAPI-stained image.

Table S4 details the keypoint generation when further improving the condition of nucleus polygon matching by using the minimum overlap threshold value of 0.92 on 12 human kidney Xenium samples using the FF preservation method. We also compare the image alignment performance between the automatic method of Xenium-Align and the method of manually placing keypoints in Xenium Explorer software in three samples, 3775, 40610, and 40775. Moreover, 112, 229, and 234 keypoints were manually placed during image alignment in Xenium Explorer for these samples, respectively. To implement image alignment in each sample, it took several hours of labor to annotate these keypoints, and they were uniformly distributed across different regions. Figure S4 shows the comparison results of image alignment in the randomly selected region between Xenium-Align and manual placement in Xenium Explorer software for the three samples.

Table 1 shows two samples (F59 and 40610) that can generate more than 30 keypoints; three samples (20012, 40440, and 40775) that output a number of keypoints (between 20 and 30); and less than 20 keypoints can be obtained from seven samples (26429, 36816, 3723, 3775, 3781, 38111, and 5582). Most of the samples used the Cellpose model to implement nucleus segmentation, and the StarDist model was used on only two samples (3775 and 38111). As the widths in the H&E image and the DAPI-stained image were much shorter than the lengths, the crop radius sizes were set to half of the width of the DAPI-stained image to obtain the search region that included enough cell nuclei in three samples (40440, 40775, and 5582). The crop ratio of the minimum value between the width and length of the DAPI-stained image was set to 0.06 on two samples, 38111 and 3775, due to the shapes of the H&E image and the DAPI-stained image being nearly square. As for the running time, only one sample, 38111, was more than 30 h; this is due to its large epoch number of 15 (See Section 3.2). Half samples (F59, 20012, 36816, 40440, 40610, and 5582) were able to automatically conduct image alignment within 20 h.

As shown in Table 2, all accurate keypoints could be obtained using our proposed identification method on ten samples, except for two samples, F59 and 3781. These two samples had one pair of false keypoints in their respective output results. When evaluating the threshold number of false keypoints, randomly setting 30 percent of the total number of false keypoints can obviously influence the performance of image alignment in most cases. Four samples (3781, 38111, 40440, and 40775) and one sample (20012) obtained obviously poor image alignment when randomly setting 20 percent and 10 percent of the total number of false keypoints, respectively. We also evaluated the performance of image alignment in cases where random subsets of keypoints were selected from the keypoints generated by Xenium-Align for these samples. The selection numbers were fewer than the total number of keypoints, and in intervals of five (such as 35, 30, 25, and so on). As the number of keypoints decreased, there was no obvious impact on the overall performance of image alignment. Performance in certain regions with a lack of keypoints was affected. Based on the requirement of at least three keypoints as input in Xenium Explorer, within a given region, the greater the number of matched keypoints, the better the results obtained for image alignment.

As shown in Figure 2A, our proposed keypoint identification method can achieve good image alignment performance in Xenium Explorer software. The nucleus boundaries in red are from the Xenium DAPI-stained image. They accurately describe the boundaries of cell nuclei in the H&E image. This indicates that cell nuclei in the H&E and DAPI-stained images can completely overlap. As shown in Figure 2B, when we randomly set the threshold number of false keypoints in the keypoint file of Xenium-Align, after using it to implement image alignment in Xenium Explorer software, the nucleus boundaries are moved and cannot describe the boundaries of cell nuclei in the H&E image. The cell nuclei in the H&E image and the DAPI-stained image are far from each other, which obviously influences the performance of image alignment. It also demonstrates how many false ones in the keypoints lead to poor image alignment. As shown in Figure 3, 36 generated keypoints are uniformly distributed across the different overall matched regions of the H&E image and DAPI-stained image, even if a single pair of false keypoints is present. This spatially uniform dispersion of keypoint placement contributes to the good image alignment performance. As shown in Figure 4, the accurate keypoints are placed in the same cell nuclei of the DAPI-stained image and the H&E image. The placements are exactly at the center points of both cell nuclei. As shown in Figure S3, the false keypoints are placed in different cell nuclei, resulting in mismatched positions between the DAPI-stained image and the H&E image. For the current placement in the DAPI-stained image, the accurately matched placement should be located in the red box of the H&E image.

As shown in Table S4, when the condition of nucleus polygon matching is improved in Xenium-Align, 11 samples obtain all accurate keypoints, except for one sample, 3781. Compared with the case of setting the average overlap threshold to 0.9, using a minimum overlap threshold of 0.92 increases the accuracy of identifying matched keypoints, but the computational time becomes longer. It takes more than 45 h to generate the keypoints in most cases. Based on the stricter matching condition, a smaller number of keypoints can be kept by the processing of nucleus polygon matching in each epoch. The longer computational time is based on the higher probability of identifying the accurate keypoints that are placed in the same cell nuclei of the DAPI-stained image and the H&E image. As shown in Figure S4, while the manual method places far more keypoints than Xenium-Align, Xenium-Align achieves the same good image alignment performance in the randomly selected region of the three samples. They illustrate only minor differences in alignment performances. The cell nuclei can be well-aligned between the H&E image and the DAPI-stained image in both methods. However, the method of manual placement requires a large amount of labor costs for defining the accurate keypoints. The matched keypoint file can be automatically generated by Xenium-Align, and it obtains image alignment results of comparable accuracy within the Xenium Explorer software.

### 3.2. Description of Image Processing and Multi-Directional Enhanced Image Assessment

**Figure 5:**
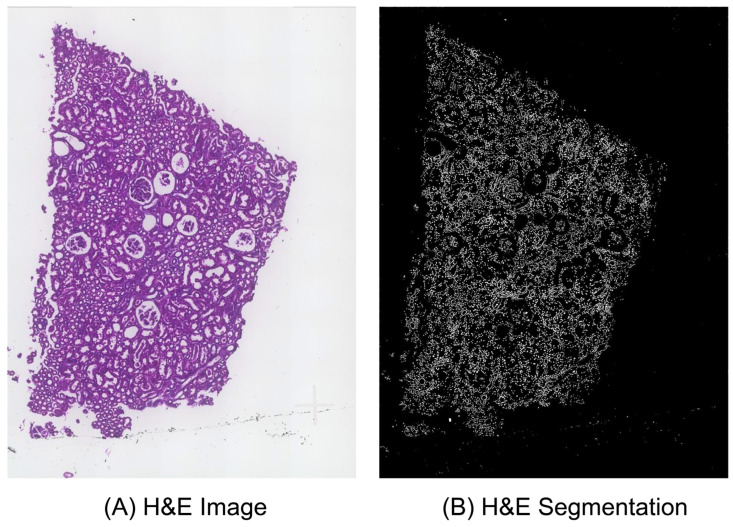
Comparison results between the H&E image and its nucleus segmentation image for sample F59; (**A**) shows the original H&E image; (**B**) shows the nucleus segmentation image.

**Figure 6:**
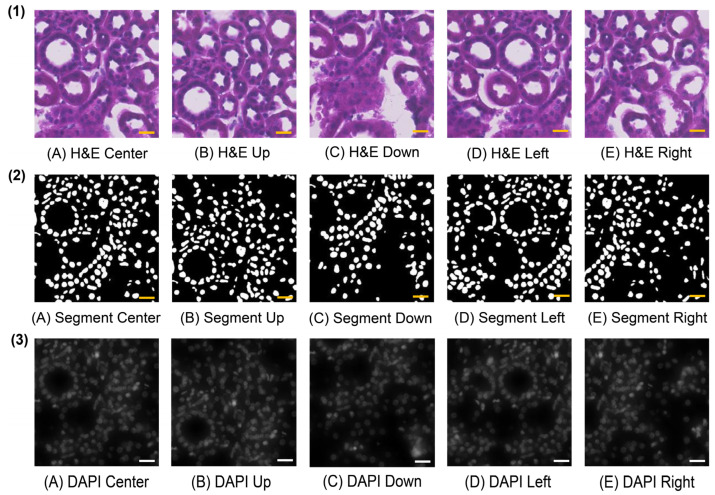
Comparison results of cropped patches from the (**1**) H&E image, (**2**) H&E segmentation, and (**3**) DAPI-stained image in five moving directions, that is, (**A**) center, (**B**) up, (**C**) down, (**D**) left, and (**E**) right for a selected pair of cell nuclei on sample F59. The radius size of the cropped patch is 400 pixels, and the moving distance from the center points is 300 pixels. The central cell in the Xenium data from the DAPI-stained image is identified by barcode *gggbgpij-1*; (scale bar, 10 μm).

Image processing provides the foundational work for identifying the matched keypoints. And the initially matched keypoints can be obtained after multi-directional enhanced image assessment. Figure 5 shows the comparison results between the whole H&E image and its nucleus segmentation image for sample F59. Tables S2 and S3 detail the hyperparameter settings and the segmentation results of the nucleus segmentation models on 12 Xenium samples using the FF preservation method. Figure 6 illustrates the comparison results of cropped patches from the H&E image, the H&E segmentation, and the DAPI-stained image in five moving directions for a selected pair of cell nuclei, in which the central cell in the Xenium data from the DAPI-stained image is identified by barcode *gggbgpij-1*. Figure S5 shows the visualization results of the search region in the DAPI-stained image for the selected pair of cell nuclei. Figure S6 shows the results of multi-directional enhanced image assessment in each epoch and the number of epochs using Xenium-Align on the 12 Xenium samples.

As shown in Figure 5, the overall layout of the segmented cell nuclei label image is generally consistent with the original H&E image. The cell nuclei labels in the segmentation image represent the actual cell locations in the H&E image. As shown in Table S3, the number of output segments is more than 60 percent of the total cell numbers in all cases. The segment ratio of sample 40775 is even larger than 75 percent. Because Cellpose is the first choice for implementing nucleus segmentation in Xenium-Align, the segment ratios are 28 and 40 percent of the Cellpose model on samples 3775 and 38111, respectively. We implemented nucleus segmentation using the StarDist model and obtained better segment ratios (more than 65 percent on these two samples). As illustrated in Figure 6, based on the center point in the selected nucleus, three center image patches can be cropped from the H&E, H&E segmentation, and DAPI-stained images. After moving in four directions (up, down, left, and right) with the same distances from the center coordinates, five image patches can be obtained in three images, respectively. The overall layouts are highly consistent between H&E patches, H&E segmentation patches, and DAPI-stained patches. The largest PSNR values between H&E segmentation and DAPI-stained images can be obtained in the respective rankings from five directions (center, up, down, left, and right). The locations of cell nuclei and their positional distributions in the H&E image can be shown by the H&E segmentation images. The image patches of five moving directions capture the surrounding structure and image features of the central nucleus, facilitating reliable matching between the H&E image and the DAPI-stained image. After selecting one nucleus from the H&E image, a corresponding search region in the DAPI-stained image can be obtained.

Figure S5 shows a search region including 2060 cells; the matched cell with barcode *gggbgpij-1* is in the red box. As shown in Figure S6, two samples, 3781 and 38111, require over ten epochs to output keypoints by multi-directional enhanced image assessment. In each epoch, fewer than 12 keypoints can be generated in most cases, which results in a longer computational time. It also indicates the limited consistency degree between the segmented H&E image and the DAPI-stained image. Two samples, 26429 and 3775, require four and five epochs, respectively, with each epoch generating over ten keypoints. Four samples (F59, 3723, 40610, and 40775) and one sample (40440) conduct multi-directional enhanced image assessment in two epochs and three epochs, respectively. Three samples, 20012, 36816, and 5582, only have one epoch, generating more than 40 keypoints, resulting in a shorter computational time within ten hours. These three samples exhibit more matched patches between the segmented H&E image and the DAPI-stained image, with the same top-1 ranking in image assessment across the five moving directions.

### 3.3. Effectiveness of the Delaunay Triangulation Graph and Nucleus Polygon Matching

There were some matched keypoints with mistaken placements in the output after image processing and multi-directional enhanced image assessment. We conducted case studies to show the functional utility of Delaunay triangulation graphs and nucleus polygon matching. Figure S7 visualizes the mistaken placements that were removed by Delaunay triangulation graph matching in Xenium Explorer on sample 20012. Figure S8 shows the Delaunay triangulation graphs before and after matching using the keypoint placements on the DAPI-stained image and the H&E image. Figures S9 and S12 visualize the two cases of mistaken keypoint placements that were removed using nucleus polygon matching in Xenium Explorer on sample 38111. Figures S10 and S13 visualize the specific cropped patches of the H&E image and the DAPI-stained image when removing mistaken keypoint placements using nucleus polygon matching. Figures S11 and S14 show the cell nuclei polygons of two keypoint placements on the DAPI-stained image and the H&E image. Table S5 details the hyperparameter settings of Xenium-Align in cases of mistaken keypoint placements on two samples, 20012 and 38111.

As shown in Figure S7, the ground truth nucleus of the H&E image within the red box is to the right of the current placement. There is no corresponding nucleus on the side of the DAPI-stained image for the current H&E placement. Keypoint 3 in the H&E image lacks a corresponding ground truth match in the DAPI-stained image. Therefore, as shown in Figure S8A, the built Delaunay triangulation graphs of the H&E image and the DAPI-stained image highlight the erroneous keypoint 3, which is marked in red boxes. The edges of keypoint 3 in the two graphs are different from each other because they are placed in mismatched positions, and the distances between keypoint 3 and other keypoints are inconsistent. As shown in Figure S8B, after Delaunay triangulation graph matching, keypoint 3 can be removed as an outlier, and the resulting two graphs have a consistent topology, with the same nodes and edges.

As shown in Figure S9, paired keypoint 3 is mistakenly placed at mismatched locations in Xenium Explorer on sample 38111. As shown in Figure S10, the area of the nucleus boundary in the H&E image is significantly larger than the area of the nucleus boundary in the DAPI-stained image. The cell nuclei in the H&E image and the DAPI-stained image cannot be overlapped well. The center points in these two cell nuclei that are used as the coordinates of keypoint placements deviate from each other. The two cell nuclei polygons of mistaken keypoint 3 from the DAPI-stained image and H&E image are illustrated in Figure S11. These mistaken placements of mismatched cell nuclei can be removed using nucleus polygon matching based on the average overlap index of 0.755, which is lower than the overlap threshold of 0.9. The other case of mistaken keypoints in Xenium Explorer on sample 38111 is shown in Figure S12. Figure S13 details the differences in cropped patches between the DAPI-stained image and the H&E image. Several cell nuclei are merged together after nucleus segmentation in the H&E image, resulting in the nucleus being much larger than the identified nucleus of the DAPI-stained image. As illustrated in Figure S14, the shapes and sizes of the two cell nuclei polygons from the DAPI-stained image and H&E image are very inconsistent. These two mismatched cell nuclei can be filtered out using nucleus polygon matching based on the average overlap index of 0.847, which is also lower than the overlap threshold of 0.9.

### 3.4. Application of Xenium-Align to the New Test Dataset

To further validate our proposed keypoint identification method on the new test dataset, we implemented Xenium-Align on two human kidney samples and one human skin Xenium sample using the FFPE preservation method to generate a keypoint file for automatic image alignment in Xenium Explorer software. Table S6 shows the hyperparameter settings and the results of keypoint generation on three Xenium samples (kidney cancer, non-diseased kidney, and non-diseased skin). Figure S15 illustrates the keypoint placements in Xenium Explorer on the kidney cancer sample.

As shown in Table S6—because the hyperparameter that controls the minimum number of generated keypoints is set to 10–11, 15, and 13 keypoints can be output by Xenium-Align for the cancer and non-diseased kidney samples, as well as non-diseased skin samples, respectively. The matching of the placed keypoints between the DAPI-stained image and H&E image in image alignment is 100 percent accurate for these three samples. As for the running times, around 28.4, 2.9, and 1.4 h are taken to generate the fully accurate keypoints for these three samples, respectively. The large epoch number of 51 on the cancer sample indicates that the number of identified keypoints that meet the conditions of multi-directional enhanced image assessment, Delaunay triangulation graph, and nucleus polygon matching is small, even though no keypoints are generated for the 100 sampled cells from the segmented H&E image in some cases.

As illustrated in Figure S15, 11 pairs of keypoints are placed in different matched locations between the DAPI-stained image and H&E image in Xenium Explorer on the kidney cancer sample. The locations of keypoint clusters are predominantly in the middle regions of both images. Compared with other regions, the middle regions contain relatively fewer cell nuclei. The background color areas (with black in the DAPI-stained image and white in the H&E image) are larger. Color distinctions between the cell nuclei and backgrounds are more obvious, which contributes to identifying the matched patches between the two images by the PSNR index with enhanced assessment in five directions. When the sampled cells of the H&E image are in the non-middle regions, it is more difficult to identify matched cell nuclei from the search region of the DAPI-stained image and output the accurate keypoints in each epoch by Xenium-Align.

Overall, the experimental results of two human kidney samples and one human skin sample with the FFPE preservation method demonstrate Xenium-Align’s generalizability to the new test dataset, its applicability to Xenium samples of different organs, and its effectiveness in saving labor costs for automatic image alignment.

## 4. Discussion

This study proposes a keypoint identification method, Xenium-Align, which can generate an accurate keypoint file to automatically conduct image alignment between imported H&E images and DAPI-stained images for Xenium Explorer. The framework of Xenium-Align comprises five function modules, namely, image processing, multi-directional enhanced image assessment, the Delaunay triangulation graph, nucleus polygon matching, and keypoint file generation.

In image processing, the H&E image is automatically rotated in the same direction as the Xenium DAPI-stained image, and the nucleus segmentation model is used to localize the cell nuclei. After sampling one nucleus from the segmented H&E image, a corresponding search region that includes some cells in the DAPI-stained image can be set to search for the matched nucleus. We crop the image patches for the prepared cell nuclei in two images. In multi-directional enhanced image assessment, we apply the widely used image quality index of PSNR to evaluate the similarity between the cropped patches of the H&E image and the DAPI-stained image. The cropped patches in five directions (center, up, down, left, and right) are used to represent the center nucleus. And the same top-1 ranking cell nuclei of the DAPI-stained image for the sampled H&E nuclei in five directions are used as the initially matched keypoints for the next steps. In the two matching methods, we filter out the outliers from the aspects of topology consistency between two graphs and overlap degrees between already matched nuclei, respectively. The method of evaluating edge consistency is used to keep two graphs with the same nodes and edges in Delaunay triangulation matching. Nucleus polygon matching applies average or minimum overlap indexes to evaluate whether two nuclei come from the same cell. In keypoint file generation, the accurately matched keypoints are used as the input. We generate the CSV file with spatial coordinates of keypoints as the available format for importing into Xenium Explorer software.

The 15 human samples from two Xenium datasets, in which 12 human kidney samples use the FF preservation method, while two human kidney samples and one human skin sample are profiled from FFPE tissue, are used to test our proposed keypoint identification method for evaluating the performance of automatic image alignment in Xenium Explorer software. Xenium-Align can generate all correct keypoints on 14 Xenium samples from two human organs. The experimental results demonstrate that our proposed method can automatically generate accurate keypoints to obtain effective image alignment performance. The proposed keypoint identification method addresses the labor-intensive issue of keypoint placement manually during image alignment. It not only assists in the existing studies, including visualizing image alignment between the H&E image and the DAPI-stained image, but also actually saves labor costs of manual operation and improves the usage efficiency of Xenium Explorer software.

Image processing is the basis of our proposed keypoint identification method, which provides the cropped patches of candidate cell nuclei for image similarity assessment. The center points of each nucleus are used as the locations for keypoint placements in image alignment. They are derived from the boundary coordinates of each nucleus, which ensures placement accuracy. It helps achieve more accurate locations than manual placements because our method’s placements use the center coordinates of cell nuclei, especially in the H&E image. The segment labels of cell nuclei from the nucleus segmentation result of the H&E image truly reflect the internal color features and distribution [14], which cannot be visually captured in some cases. As long as the cell nuclei are matched correctly by the function modules in Xenium-Align, the keypoint placements can be located at the target points. Multi-directional enhanced image assessment is critical for identifying matched nuclei. It generates the initially matched cell nuclei by evaluating five rankings together. The higher the similarity between the segmented H&E image and the DAPI-stained image, the more initially matched cell nuclei can be obtained in each epoch, as observed in three samples, 20012, 36816, and 5582. These preliminary matches are used as the input to the next processing steps of the two matching methods, which are Delaunay triangulation graph matching and nucleus polygon matching [42,45].

The global and local matches between the keypoints of the H&E image and the DAPI-stained image can be assessed by Delaunay triangulation graph matching and nucleus polygon matching, respectively. The Delaunay triangulation graph removes the outliers by evaluating the edge consistency between graph nodes, such as the matching process, as seen in Figures S1 and S8. It keeps the consistent topology between the two graphs of the H&E image and the DAPI-stained image. Nucleus polygon matching evaluates the overlap degree from the aspects of shape and size between two cell nuclei of the H&E image and the DAPI-stained image. Because the polygons can accurately describe the contour features of cell nuclei [51], this matching method enables detailed filtering, which contributes to more accurate matches of keypoint placements on the two images. When the matching condition of the nucleus polygon is improved, more accurately matched keypoints can be obtained, but it needs a longer computational time. Delaunay triangulation graph matching and nucleus polygon matching remove the mismatched keypoints from two different perspectives, which enhances the filtering intensity and decreases the remaining room for outliers.

## 5. Conclusions

In summary, we propose a keypoint identification method, Xenium-Align, for generating an accurate keypoint file to conduct automatic image alignment between H&E images and DAPI-stained images in Xenium Explorer software. Xenium-Align can identify the matched keypoints and achieve good image alignment performance in Xenium Explorer on all 15 experimental samples. The main novelty of this study is that Xenium-Align can replace manual placement, which requires more time and costs, to automatically output a precise keypoint file and reduce unnecessary work. It can be a complementary method for more efficient and precise image alignment in Xenium Explorer. In the experimental results, there is only one mistake in the placement of the keypoint file on sample 3781, which requires further research. Future work will focus on enhancing the runtime efficiency of Xenium-Align and improving its convenience and availability in more applications across images from diverse modalities in spatial transcriptomics data.

## Figures and Tables

**Table 1:** Details about the hyperparameter settings of Xenium-Align and the results of keypoint generation on 12 human kidney samples using the FF preservation method. The Run Time column shows the computational time in hours for keypoint identification based on the segmented H&E image and the extracted DAPI-stained image. The Keypoint Number column shows the number of final generated keypoints.

Sample	Nucleus Segmentation	DAPI Search	Evaluation and Matching	Run Time	Keypoint Number
F59	Cellpose Model	crop_radius_ratio = 0.125, extracted_region_min = 50	crop_radius_pixel = 400, center_move_pixel = 300, cell_num_each_epoch = 100, overlap_ave_threshold = 0.9, keypoints_min = 15	4.915	36
20012				3.397	24
26429				22.949	18
36816				3.543	17
3723				20.949	18
3775	StarDist Model	crop_radius_ratio = 0.06, extracted_region_min = 50		26.197	19
3781	Cellpose Model	crop_radius_ratio = 0.125, extracted_region_min = 50		22.854	16
38111	StarDist Model	crop_radius_ratio = 0.06, extracted_region_min = 50		36.425	16
40440	Cellpose Model	crop_radius_ratio = 0.5, extracted_region_min = 50		15.253	21
40610	Cellpose Model	crop_radius_ratio = 0.06, extracted_region_min = 50		8.749	34
40775		crop_radius_ratio = 0.5, extracted_region_min = 50		21.559	22
5582				8.151	16

**Table 2:** Details of keypoint generation on 12 human kidney samples using the FF preservation method. N_Keypoints is the number of generated keypoints using Xenium-Align. N_Accurate is the number of accurate keypoints in the output of our proposed keypoint identification method. N_Threshold is the threshold number of false keypoints that obviously influence the performance of image alignment in Xenium Explorer software. The value in the brackets is the proportion of false keypoints to the total number of keypoints.

Sample	F59	20012	26429	36816	3723	3775
N_Keypoints	36	24	18	17	18	19
N_Accuate	35	24	18	17	18	19
N_Threshold	11 (0.3)	2 (0.1)	5 (0.3)	5 (0.3)	5 (0.3)	6 (0.3)
Sample	3781	38111	40440	40610	40775	5582
N_Keypoints	16	16	21	34	22	16
N_Accuate	15	16	21	34	22	16
N_Threshold	3 (0.2)	3 (0.2)	4 (0.2)	10 (0.3)	4 (0.2)	5 (0.3)

## Data Availability

The 12 human kidney Xenium samples using the FF preservation method are available upon request. The two human kidney samples and one human skin sample with the FFPE preservation method can be accessed at https://www.10xgenomics.com/datasets (assessed on 1 January 2025). The source code and application demo can be accessed at https://github.com/YuLin-code/Xenium-Align (assessed on 1 January 2025).

## Supplementary Materials

The following supporting information can be downloaded at: https://www.mdpi.com/article/10.3390/cells14131000/s1, Figure S1. The specific matching process of evaluating the edge consistency to make the same nodes and edges between the two graphs of the H&E image and the DAPI-stained image. (A) is the edge match processing in graph matching; (B) shows the matched edge sets of two graphs after graph matching. Figure S2. The saved format in a keypoint file available to be imported into Xenium Explorer for implementing image alignment. The coordinate values in the columns of Fixed and Alignment represent the positions of keypoints in the DAPI-stained image and the H&E image, respectively. Figure S3. Visualization result of the false keypoint placements of a certain pair of keypoints between H&E image and DAPI-stained image by our proposed identification method in Xenium Explorer software on sample F59. The ground truth placement should be located in the red box. (A) is the keypoint placement of the DAPI-stained image; (B) is the keypoint placement of the H&E image. Figure S4. Comparison result of image alignment in the randomly selected region between the automatic method of Xenium-Align and the method of manually placing keypoints in Xenium Explorer software on three samples of 3775, 40610, and 40775. (A) is the example of Xenium-Align; (B) is the example of manual placement. Figure S5. Visualization result of setting the search region for the selected cell in barcode *gggbgpij-1* from the DAPI-stained image. The search region includes 2060 cells, and the selected cell is in the red box. Figure S6. The specific number of epochs and the proportion of identified matched keypoints to the total number of sampling cells in each epoch by conducting multi-direction enhanced image assessment in Xenium-Align on 12 Xenium samples with FF preservation method. Figure S7. Visualization result of the mistaken keypoint placements that are removed by Delaunay triangulation graph matching in Xenium Explorer software on sample 20012. The ground truth matched nucleus of H&E image is in red box. (A) is the keypoint placement of the DAPI-stained image; (B) is the keypoint placement of the H&E image. Figure S8. Visualization result of the Delaunay triangulation graphs before and after matching using the keypoint placements on the DAPI-stained image and the H&E image. The mistaken keypoint placements are in red boxes. (A) are the Delaunay triangulation graphs of two images before graph matching; (B) are the Delaunay triangulation graphs of two images after graph matching. Figure S9. Visualization result of the mistaken keypoint placements that are removed by nucleus polygon matching in Xenium Explorer software on sample 38111. (A) is the keypoint placement of the DAPI-stained image; (B) is the keypoint placement of the H&E image. Figure S10. Visualization result of the specific cropped patches of the DAPI-stained image and the H&E image in the case of removing mistaken keypoint placements by nucleus polygon matching on sample 38111. The keypoint placements of cell nuclei are in red boxes. (A) is the cropped patch of the DAPI-stained image; (B) is the cropped patch of the H&E image. (scale bar, 10μm). Figure S11. Visualization result of the cell nuclei polygons of two keypoint placements on DAPI-stained image and H&E image in the case of removing the mistaken keypoint placements by nucleus polygon matching on sample 38111. The average overlap index is set as 0.9 and the calculated overlap index between (A) and (B) is 0.755. (A) is the nucleus polygon of the DAPI-stained image; (B) is the nucleus polygon of the H&E image. Figure S12. Visualization result of the mistaken keypoint placements that are removed by nucleus polygon matching in Xenium Explorer software on sample 38111. (A) is the keypoint placement of the DAPI-stained image; (B) is the keypoint placement of the H&E image. Figure S13. Visualization result of the specific cropped patches of the DAPI-stained image and the H&E image in the case of removing mistaken keypoint placements by nucleus polygon matching on sample 38111. The keypoint placements of cell nuclei are in red boxes. (A) is the cropped patch of the DAPI-stained image; (B) is the cropped patch of the H&E image. (scale bar, 10μm). Figure S14. Visualization result of the cell nuclei polygons of two keypoint placements on DAPI-stained image and H&E image in the case of removing the mistaken keypoint placements by nucleus polygon matching on sample 38111. The average overlap index is set as 0.9 and the calculated overlap index between (A) and (B) is 0.847. (A) is the nucleus polygon of the DAPI-stained image; (B) is the nucleus polygon of the H&E image. Figure S15. Visualization result of keypoint placements by Xenium-Align in Xenium Explorer software on the sample of cancer kidney. (A) is the keypoint placement of the DAPI-stained image; (B) is the keypoint placement of the H&E image. Table S1. The details about the experimental Xenium dataset with FF preservation method, in which from F59 to 5582 are 12 human kidney samples. N_Cell is the number of cells in each sample, and Condition is the corresponding state of each sample. REF means the reference or healthy kidney samples, CKD means the samples taken from the patients with chronic kidney diseases, and SLE means the samples taken from the patients with autoimmune disease of Lupus. Table S2. The details about the hyper-parameter settings of nucleus segmentation models on the H&E images of 12 Xenium samples with the FF preservation method. Table S3. The segmentation results of the nucleus image segmentation model on each sample. N_Segment is the number of segments in the output of image segmentation model and N_Cell is the number of cells in each sample, respectively. Segment_Ratio is the ratio of segment numbers to the total number of cells in each sample. Table S4. The details about the keypoints generation on 12 human kidney Xenium samples with the FF preservation method, using a minimum overlap threshold value of 0.92. N_Keypoints is the number of generated keypoints using Xenium-Align. N_Accurate is the number of accurate keypoints in the output of our proposed keypoints identification method. R_Time is the computational time in hours of keypoint identification based on the segmented H&E image and the extracted DAPI-stained image. Table S5. The details about the hyper-parameter settings of Xenium-Align in the cases of mistaken keypoint placements to show the functional utility of Delaunay triangulation graph and nucleus polygon matchings on two samples of 20012 and 38111. Table S6. The details about the hyper-parameter settings of Xenium-Align and the results of keypoint generation on two human kidney samples with FFPE preservation method. N_Epoch is the number of epochs in conducting Xenium-Align for generating keypoints. N_Keypoints is the number of generated keypoints using Xenium-Align. N_Accurate is the number of accurate keypoints in the output of our proposed keypoints identification method. R_Time is the computational time in hours of keypoint identification based on the segmented H&E image and the extracted DAPI-stained image.
