# Supplementary material for: Defining Keypoints to Align H&E Images and Xenium DAPI-Stained Images Automatically
Source: Cells. 2025 Jun 30;14(13):1000. doi: 10.3390/cells14131000 (PMC12248767; doi:10.3390/cells14131000)
Supplement: Supplementary file 1 [file cells-14-01000-s001.zip › cells-3698210-supplementary.pdf]

cells

MDPI

Supplementary Material

Defining keypoints to align H&E image and Xenium DAPI-stained image automatically: Online Supplementary File

1. Supplementary Figures

1.1. Figure S1

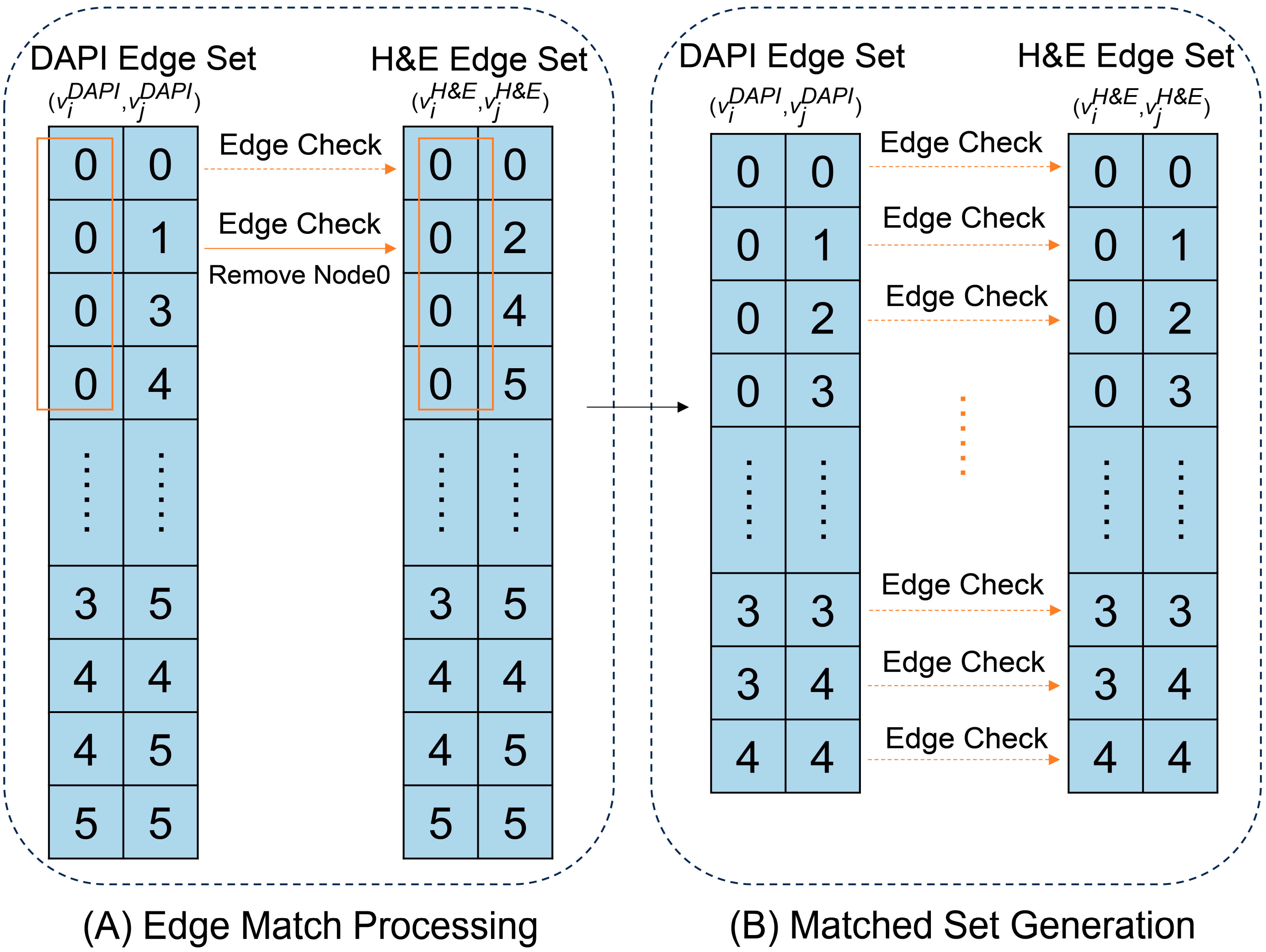

Figure S1. The specific matching process of evaluating the edge consistency to make the same nodes and edges between the two graphs of the H&E image and the DAPI-stained image. (A) is the edge match processing in graph matching; (B) shows the matched edge sets of two graphs after graph matching.

1.2. Figure S2

Fixed_X	*Fixed_Y*	*Alignment_X*	*Alignment_Y*
3562.00008616728	4048.500114889706	1813.0	1425.0
5570.0	7589.500229779412	3823.5	2503.0
8769.00017233456	4103.500114889706	1905.0	4341.5
⋮	⋮	⋮	⋮
11243.000344669115	3813.500114889706	1775.5	5731.5
7755.500057444853	7790.500344669118	3963.5	3730.0

Figure S2. The saved format in a keypoint file available to be imported into Xenium Explorer for implementing image alignment. The coordinate values in the columns of Fixed and Alignment represent the positions of key-points in the DAPI-stained image and the H&E image, respectively.

1.3. Figure S3

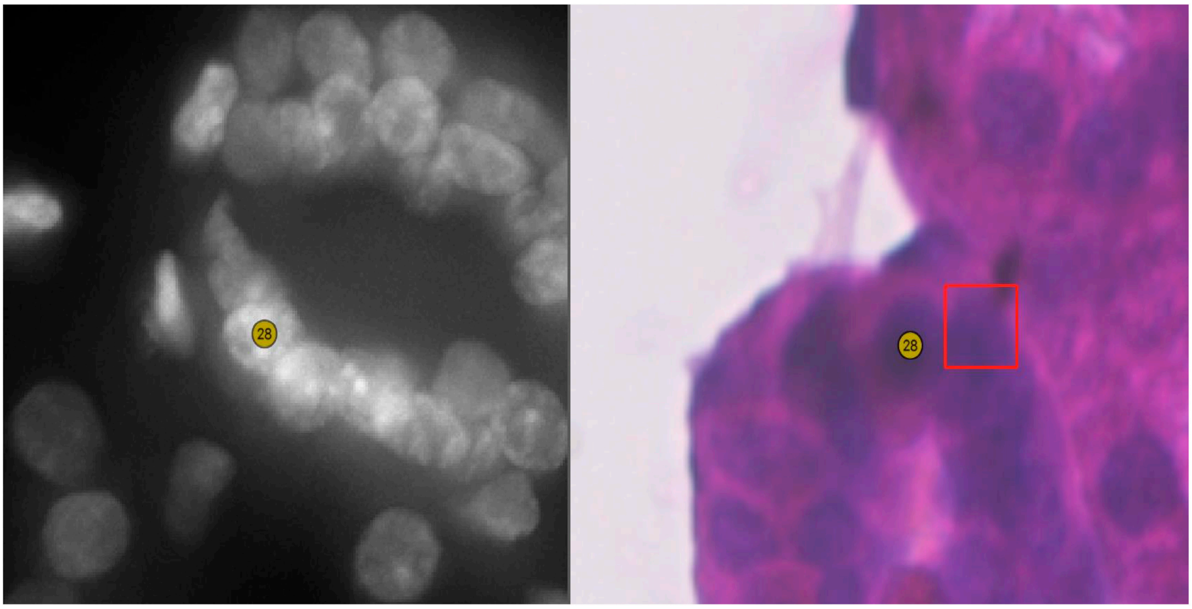

(A) DAPI Placement (B) H&E Placement

Figure S3. Visualization result of the false keypoint placements of a certain pair of keypoints between H&E image and DAPI-stained image by our proposed identification method in Xenium Explorer software on sample F59. The ground truth placement should be located in the red box. (A) is the keypoint placement of the DAPI-stained image; (B) is the keypoint placement of the H&E image.

1.4. Figure S4

1.4.1. 3775

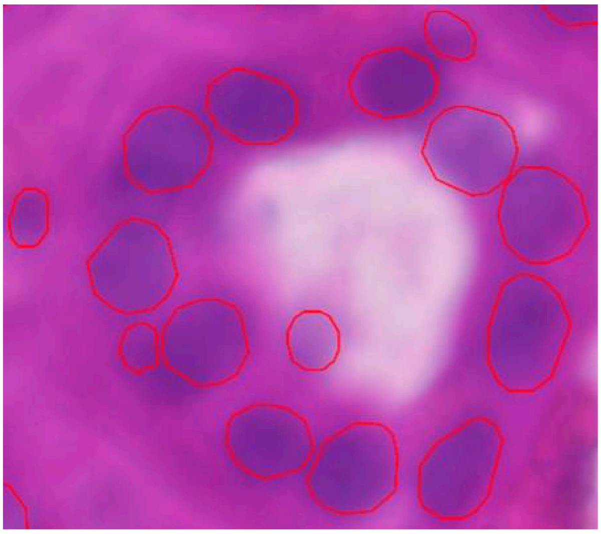

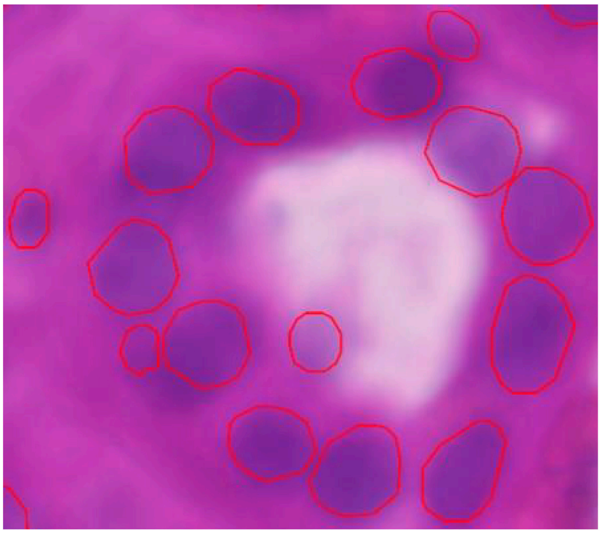

(A) Example of Xenium-Align (B) Example of Manual Placement

1.4.2. 40610

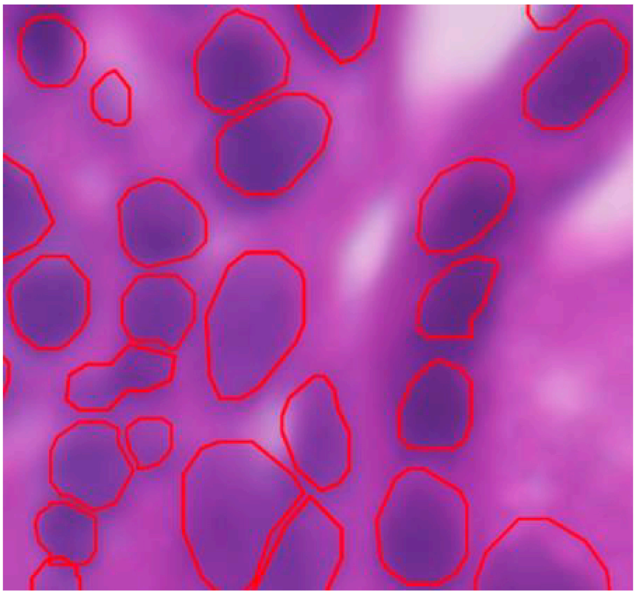

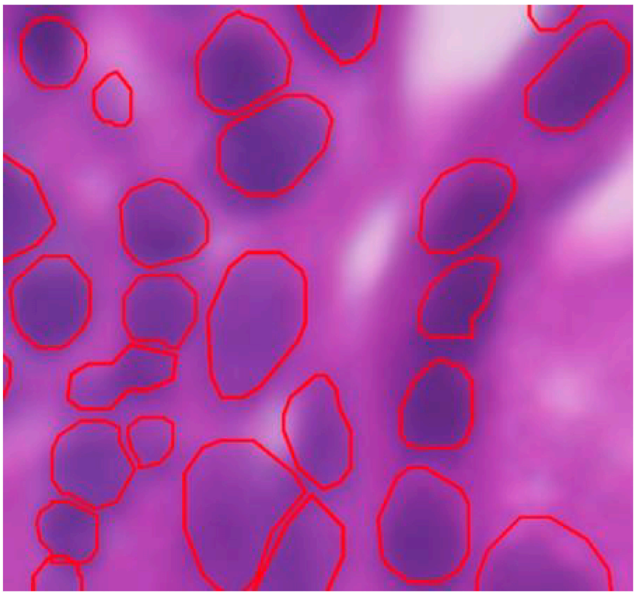

(A) Example of Xenium-Align (B) Example of Manual Placement

1.4.3. 40775

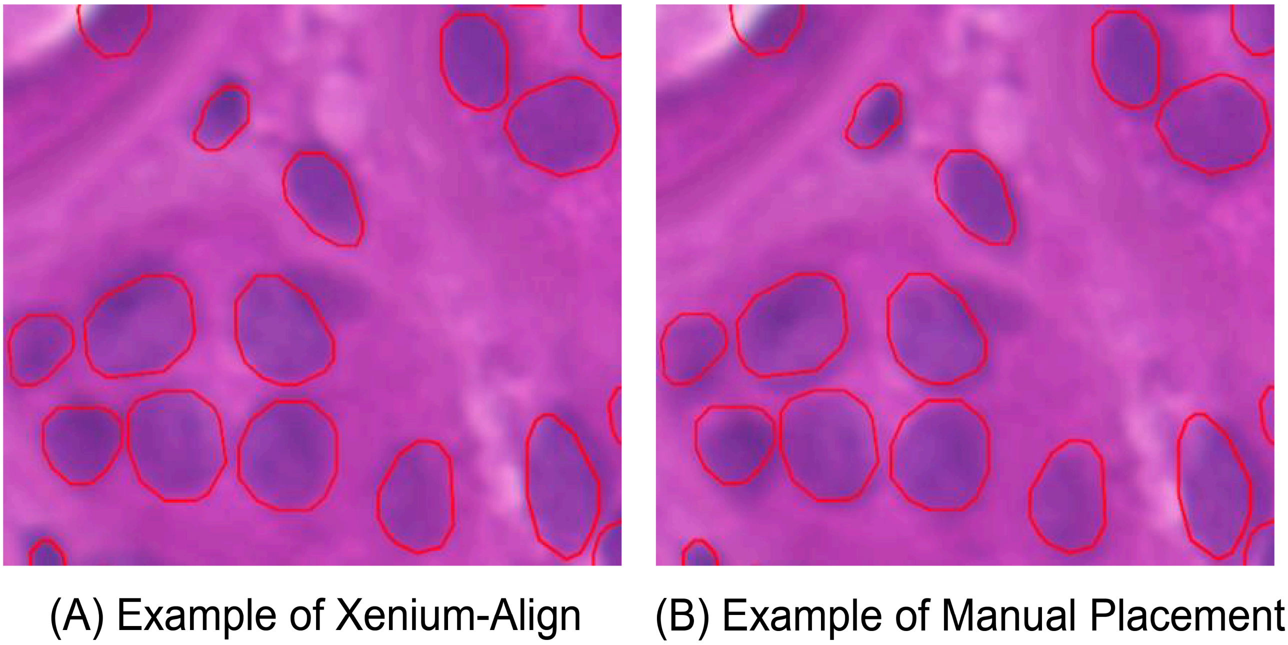

Figure S4. Comparison result of image alignment in the randomly selected region between the automatic method of Xenium-Align and the method of manually placing keypoints in Xenium Explorer software on three samples of 3775, 40610, and 40775. (A) is the example of Xenium-Align; (B) is the example of manual placement.

1.5. Figure S5

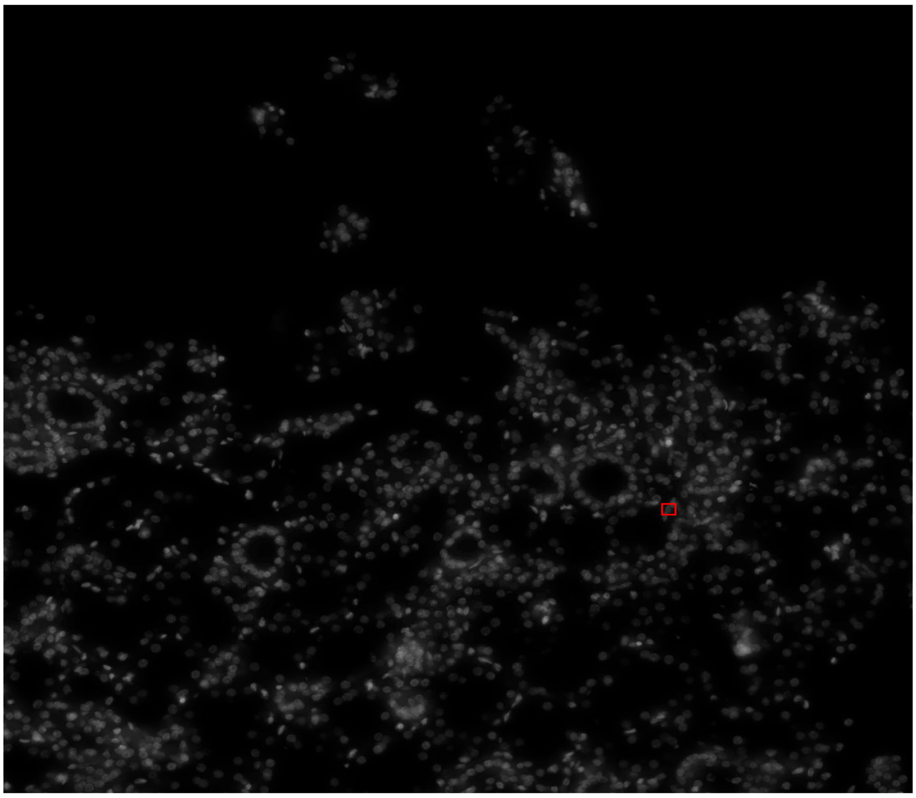

Figure S5. Visualization result of setting the search region for the selected cell in barcode *gggbgpij-1* from the DAPI-stained image. The search region includes 2060 cells, and the selected cell is in the red box.

1.6. Figure S6

1.6.1. F59

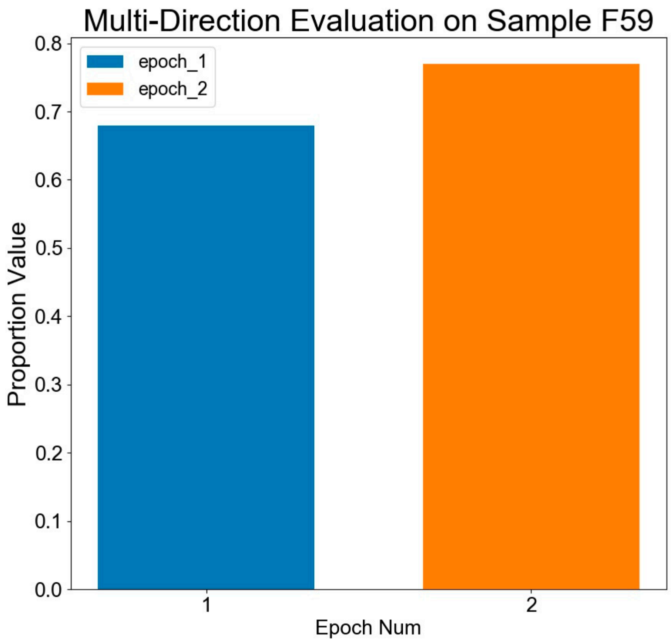

1.6.2. 26429

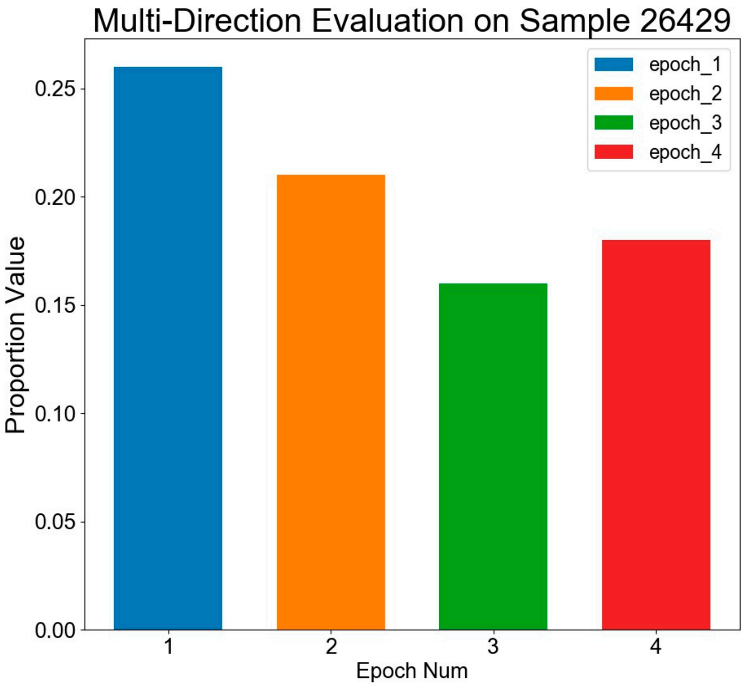

1.6.3. 3723

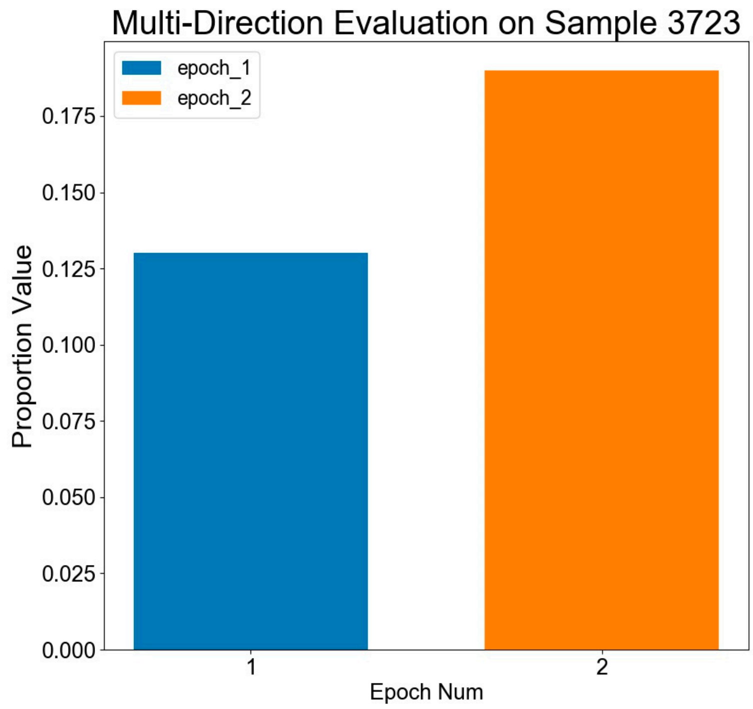

1.6.4. 3775

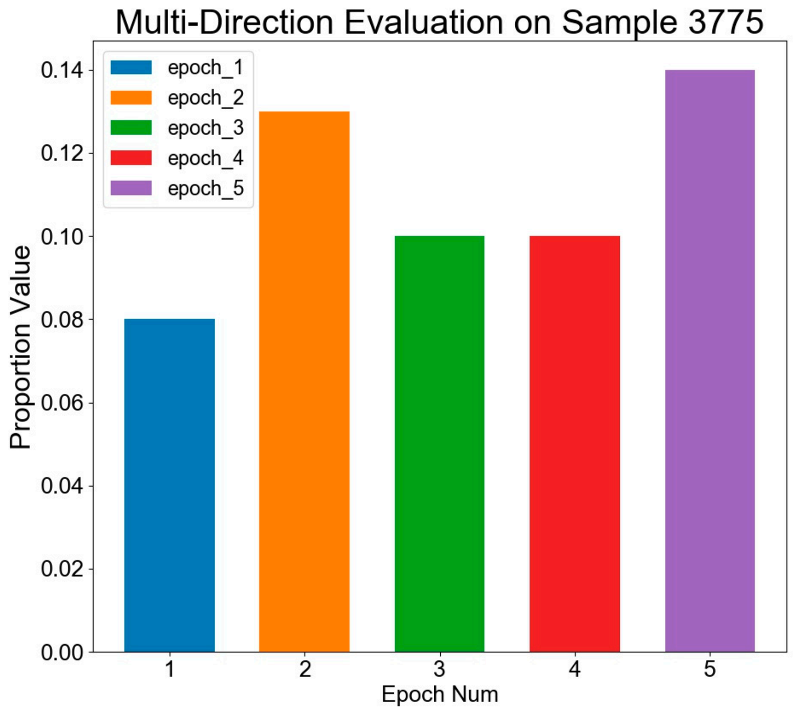

1.6.5. 3781

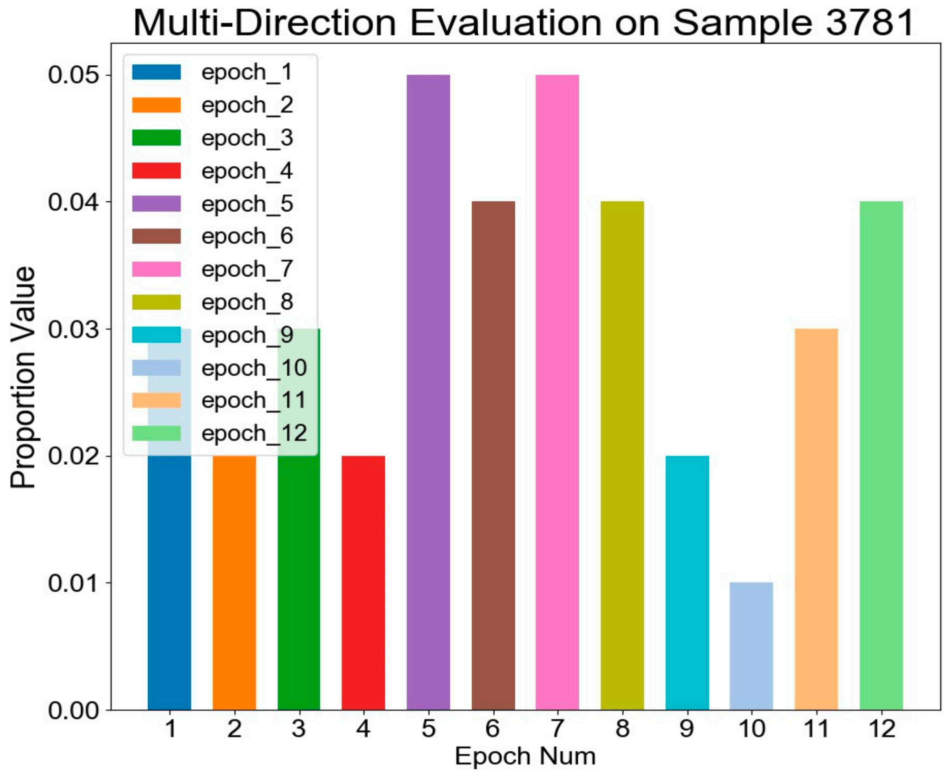

1.6.6. 38111

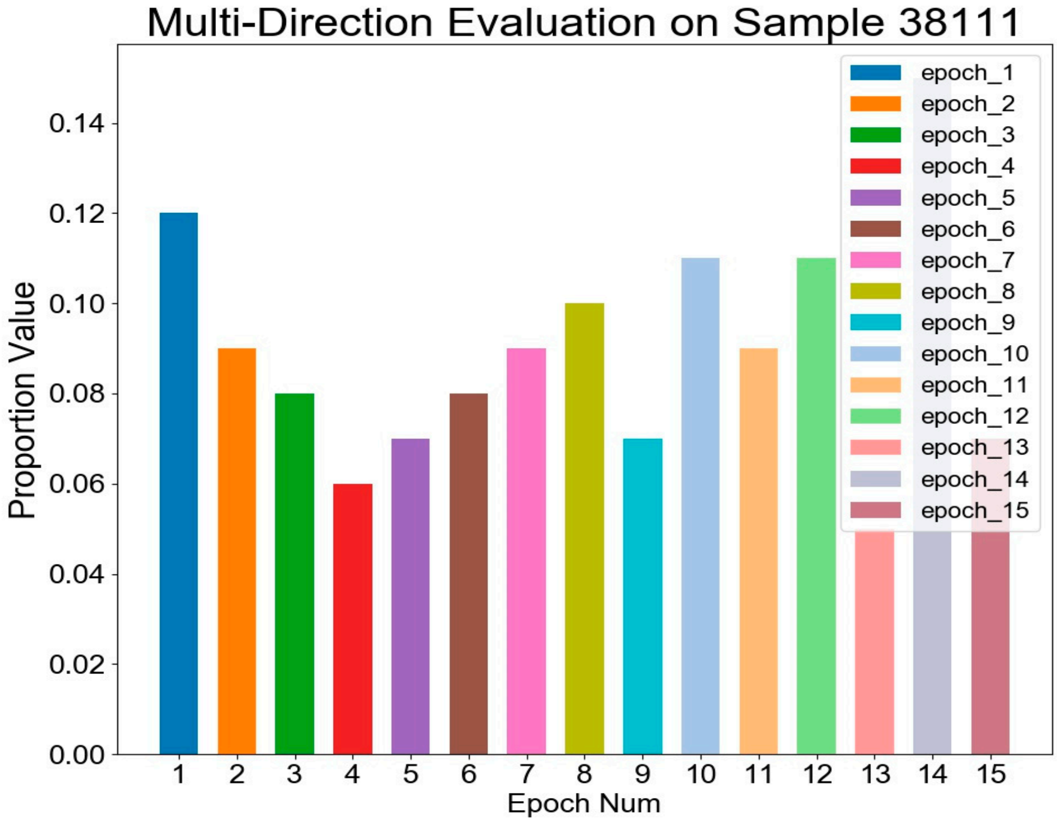

1.6.7. 40440

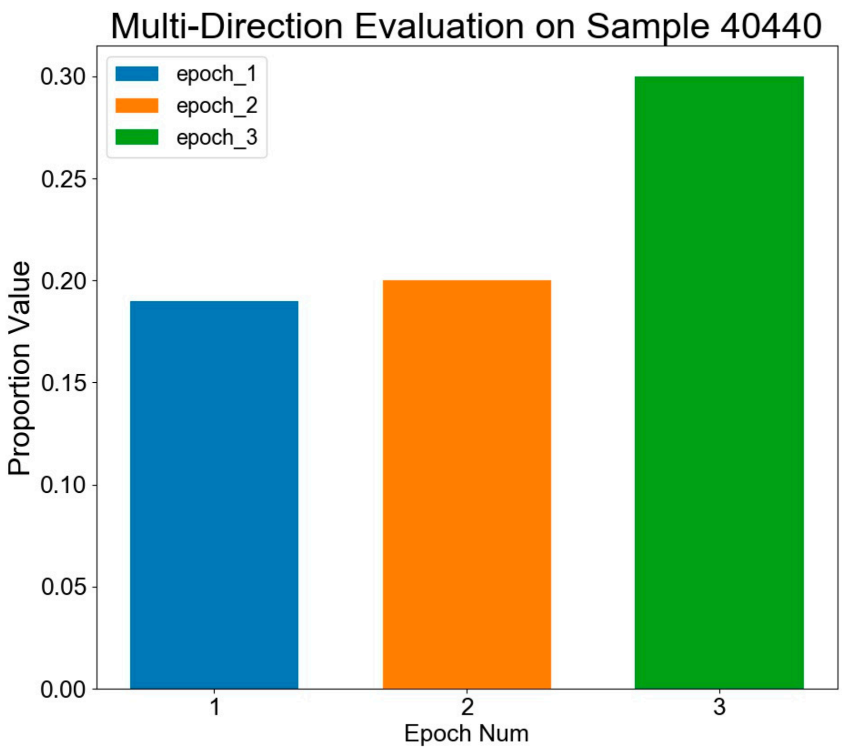

1.6.8. 40610

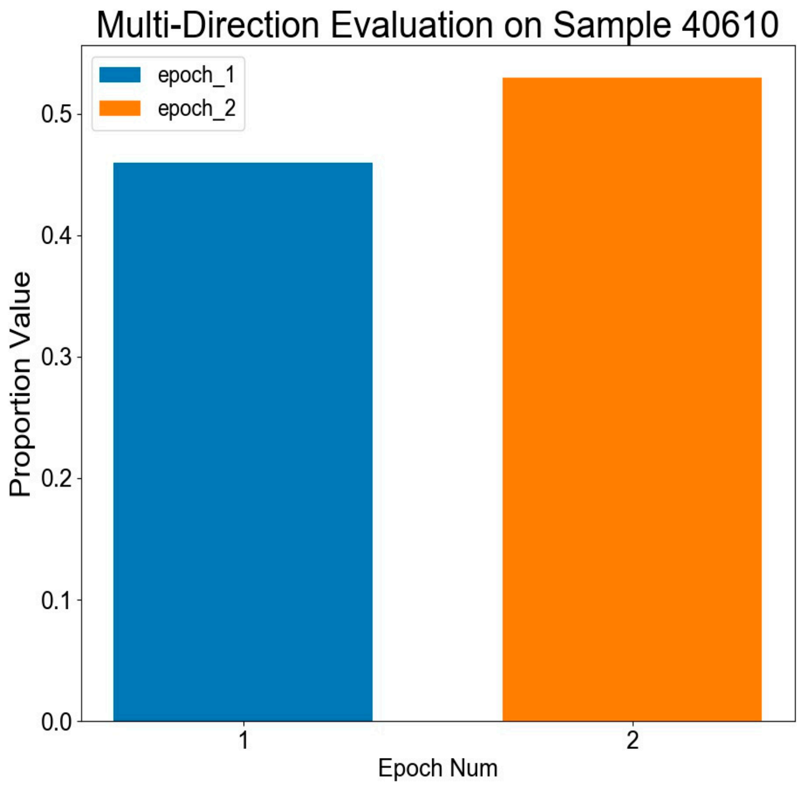

1.6.9. 40775

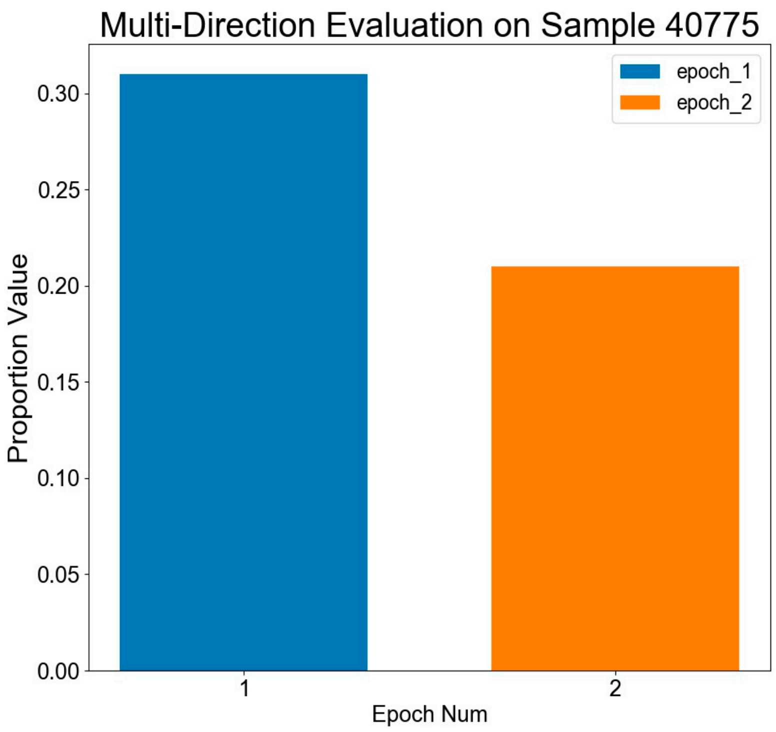

1.6.10. 20012, 36816, 5582

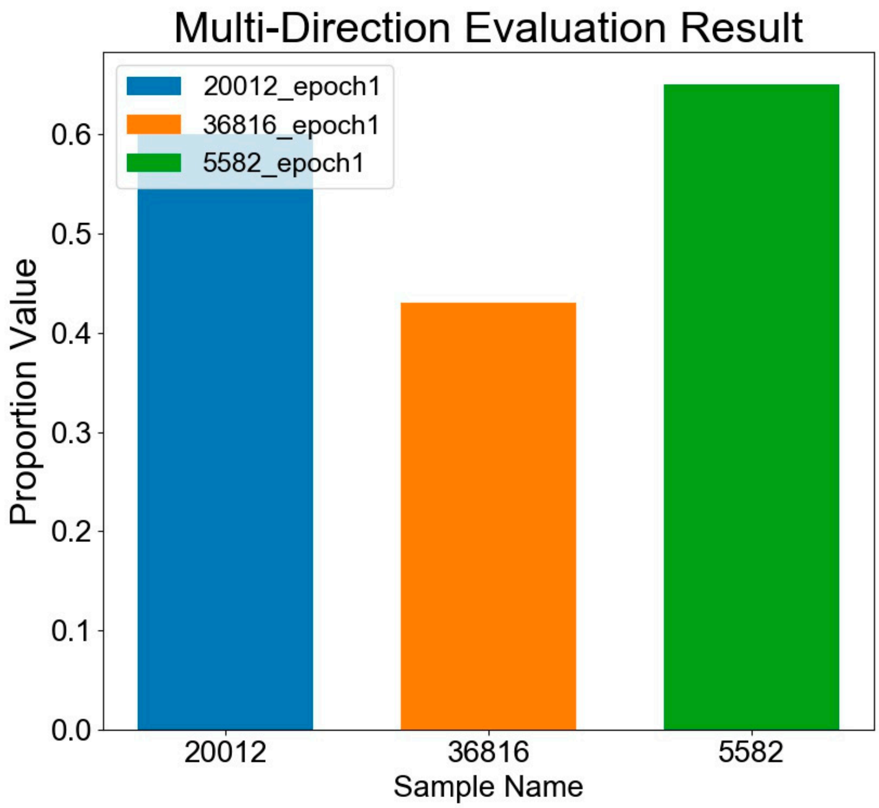

Figure S6. The specific number of epochs and the proportion of identified matched keypoints to the total number of sampling cells in each epoch by conducting multi-direction enhanced image assessment in Xenium-Align on 12 Xenium samples with FF preservation method.

1.7. Figure S7

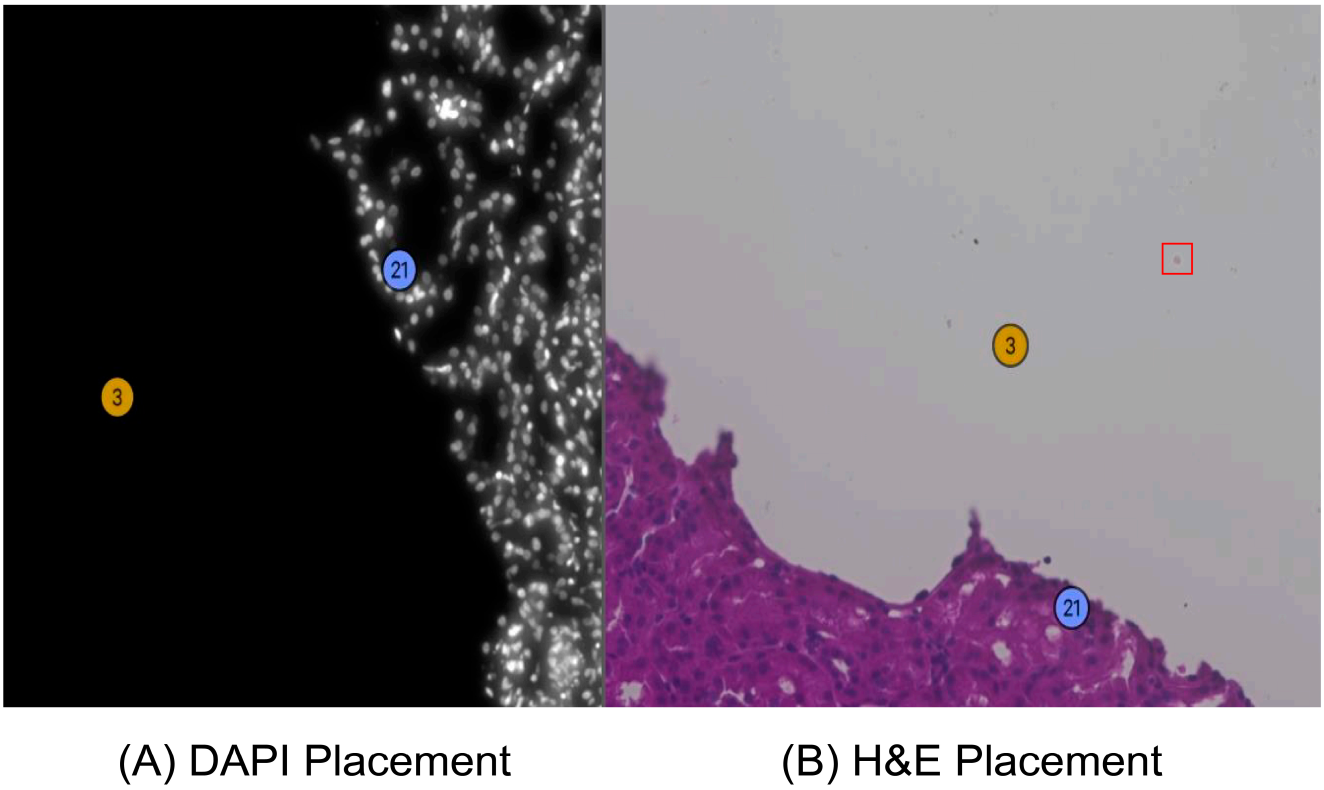

Figure S7. Visualization result of the mistaken keypoint placements that are removed by Delaunay triangulation graph matching in Xenium Explorer software on sample 20012. The ground truth matched nucleus of H&E image is in red box. (A) is the keypoint placement of the DAPI-stained image; (B) is the keypoint placement of the H&E image.

1.8. Figure S8

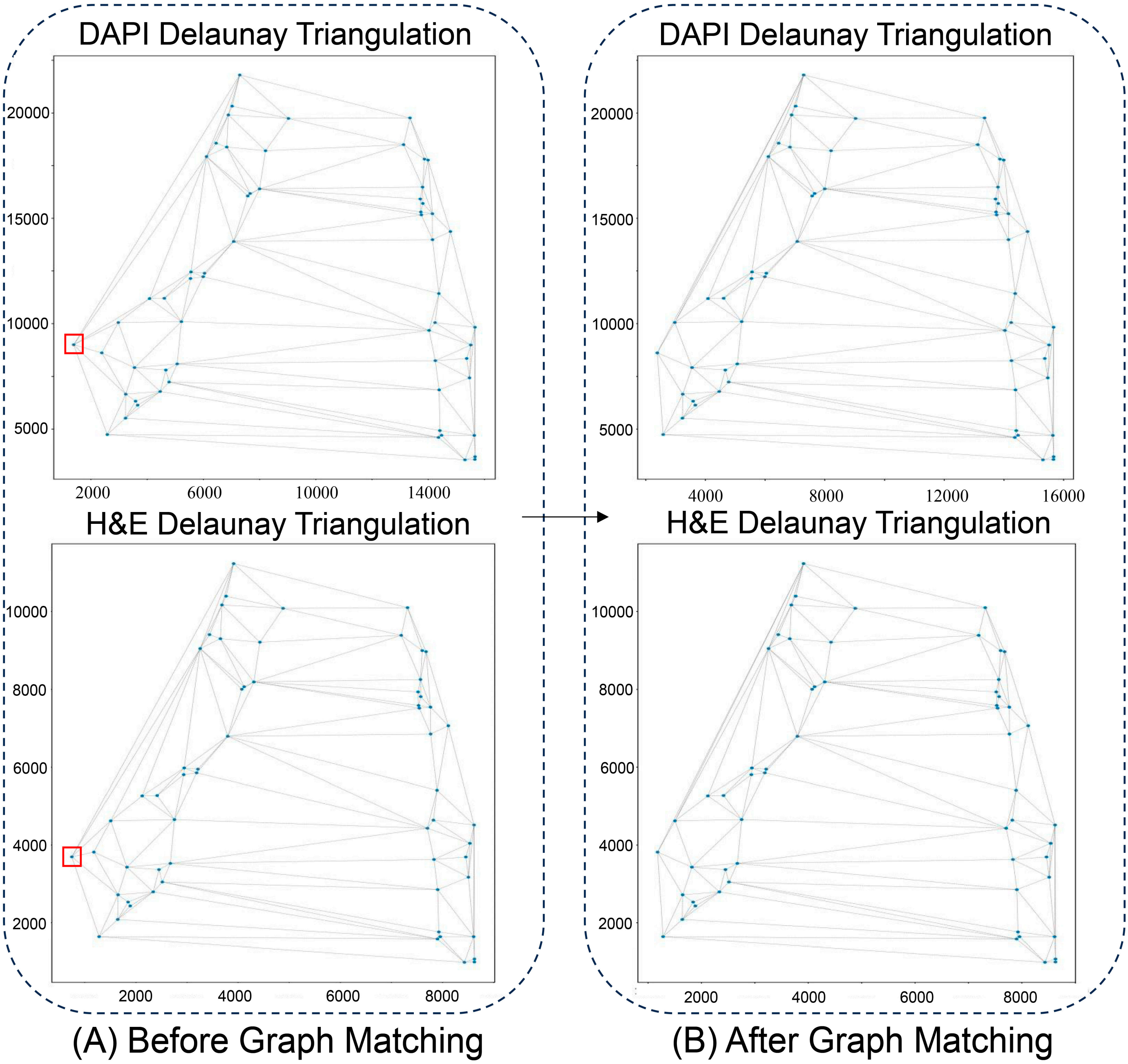

Figure S8. Visualization result of the Delaunay triangulation graphs before and after matching using the keypoint placements on the DAPI-stained image and the H&E image. The mistaken keypoint placements are in red boxes. (A) are the Delaunay triangulation graphs of two images before graph matching; (B) are the Delaunay triangulation graphs of two images after graph matching.

1.9. Figure S9

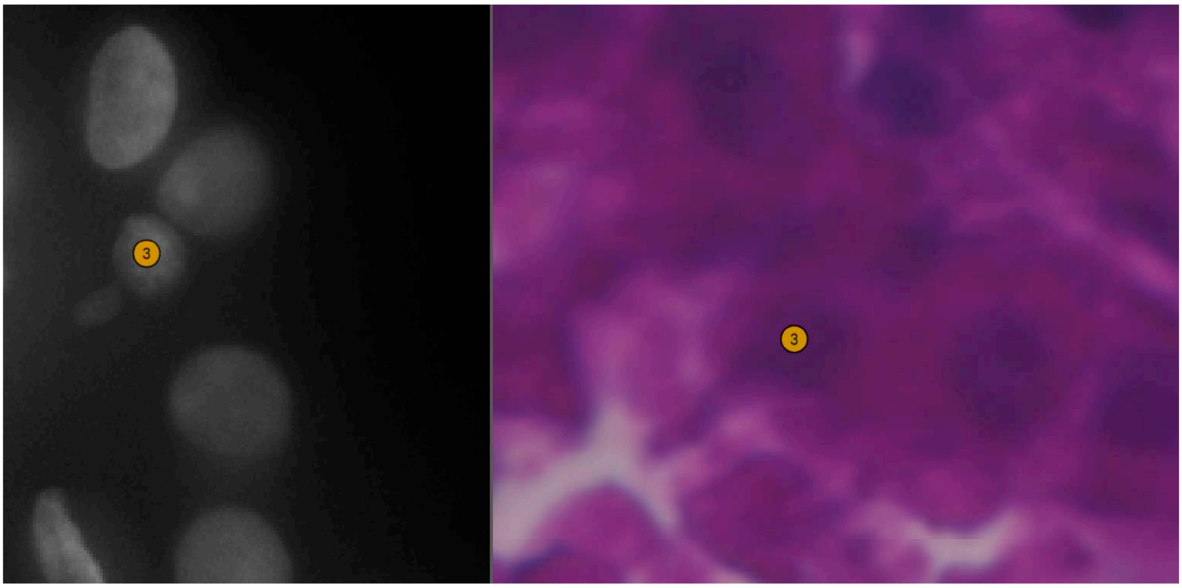

(A) DAPI Placement (B) H&E Placement

Figure S9. Visualization result of the mistaken keypoint placements that are removed by nucleus polygon matching in Xenium Explorer software on sample 38111. (A) is the keypoint placement of the DAPI-stained image; (B) is the keypoint placement of the H&E image.

1.10. Figure S10

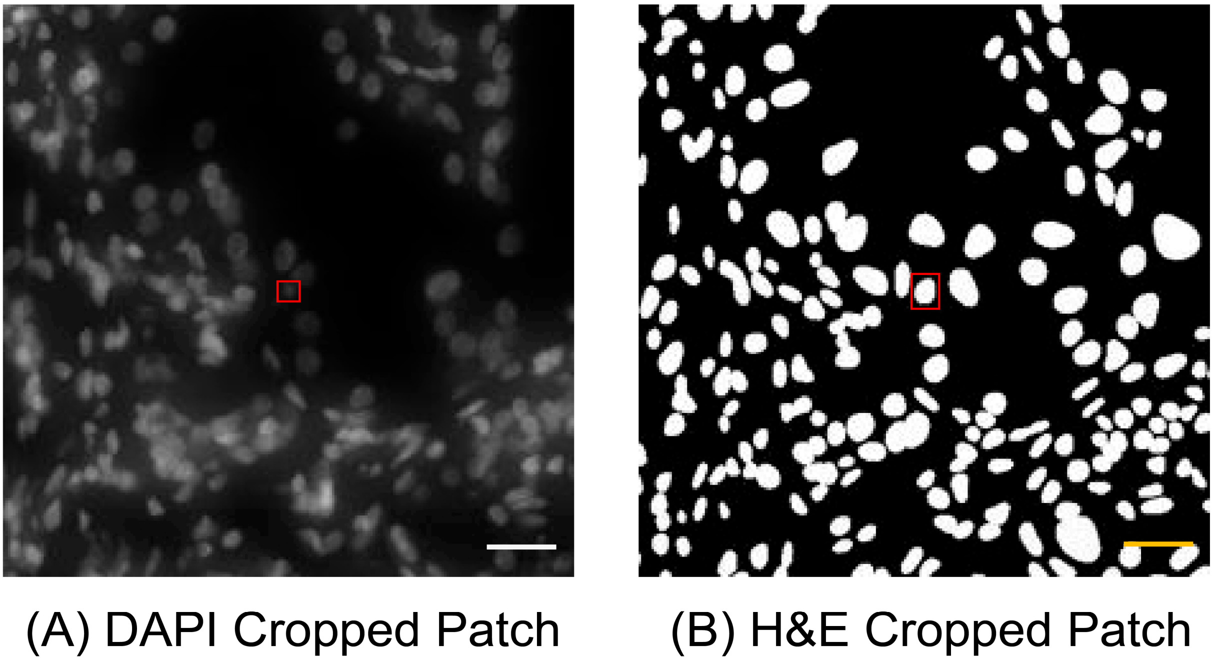

Figure S10. Visualization result of the specific cropped patches of the DAPI-stained image and the H&E image in the case of removing mistaken keypoint placements by nucleus polygon matching on sample 38111. The keypoint placements of cell nuclei are in red boxes. (A) is the cropped patch of the DAPI-stained image; (B) is the cropped patch of the H&E image. (scale bar, 10μm).

1.11. Figure S11

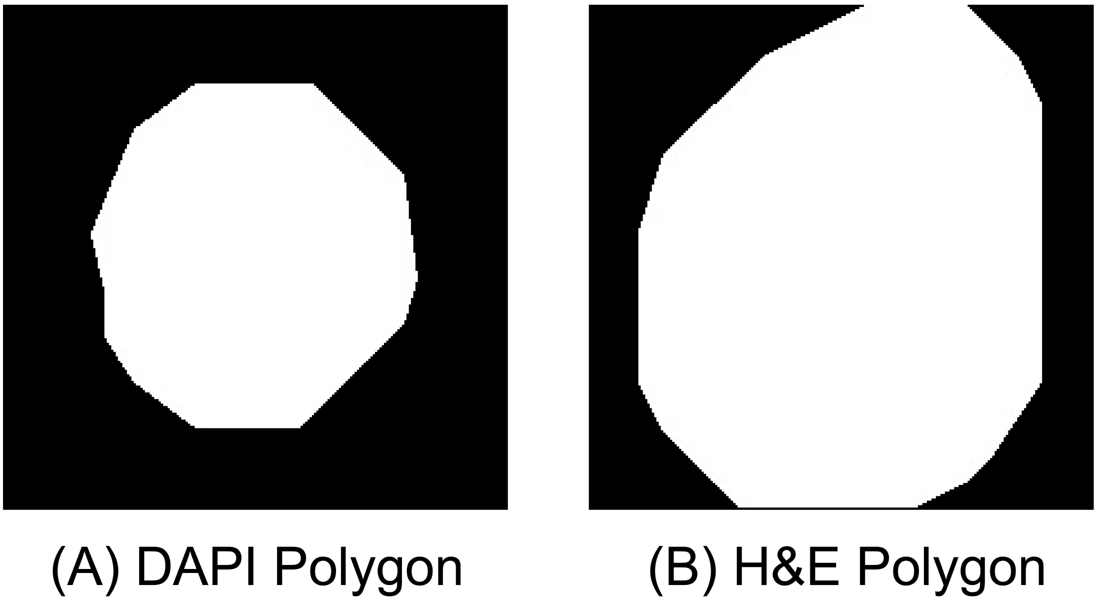

Figure S11. Visualization result of the cell nuclei polygons of two keypoint placements on DAPI-stained image and H&E image in the case of removing the mistaken keypoint placements by nucleus polygon matching on sample 38111. The average overlap index is set as 0.9 and the calculated overlap index between (A) and (B) is 0.755. (A) is the nucleus polygon of the DAPI-stained image; (B) is the nucleus polygon of the H&E image.

1.12. Figure S12

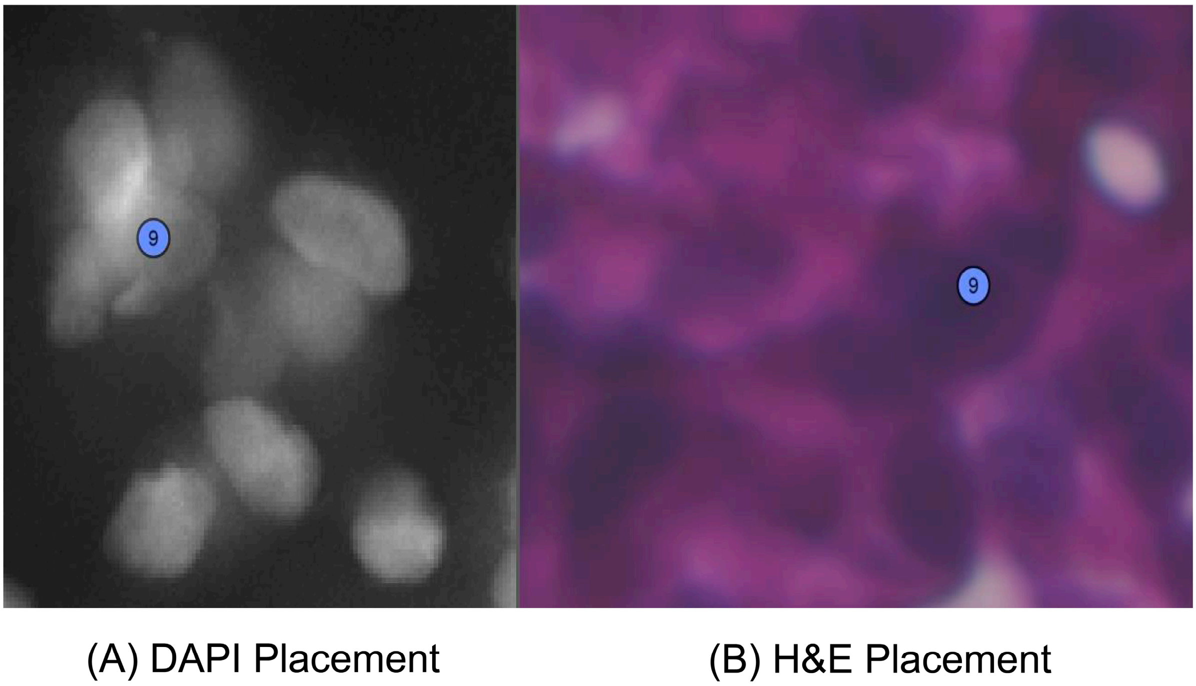

Figure S12. Visualization result of the mistaken keypoint placements that are removed by nucleus polygon matching in Xenium Explorer software on sample 38111. (A) is the keypoint placement of the DAPI-stained image; (B) is the keypoint placement of the H&E image.

1.13. Figure S13

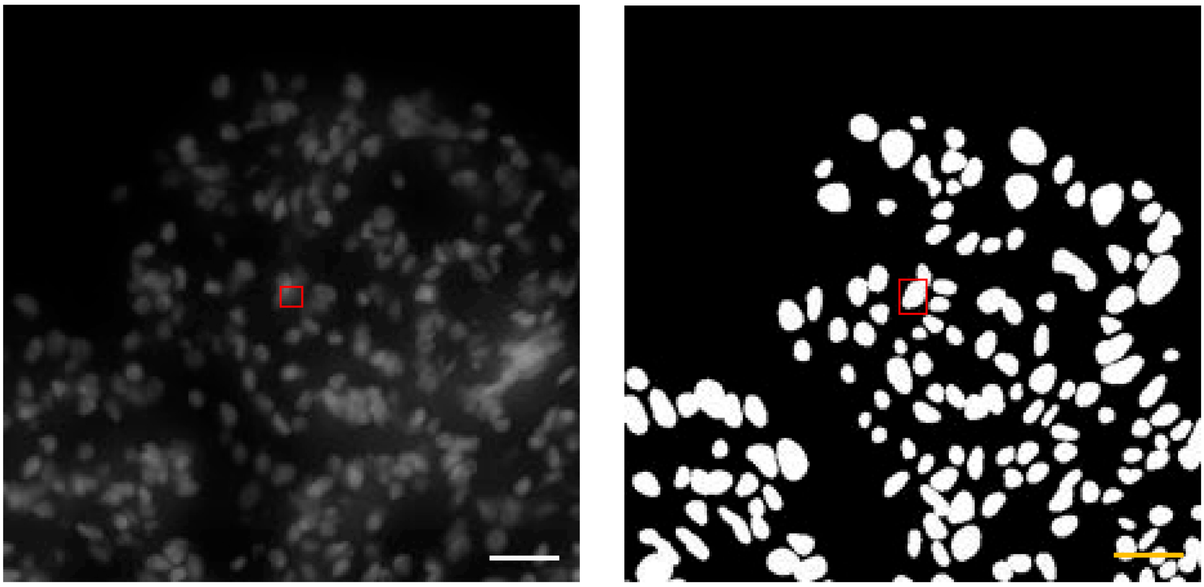

(A) DAPI Cropped Patch (B) H&E Cropped Patch

Figure S13. Visualization result of the specific cropped patches of the DAPI-stained image and the H&E image in the case of removing mistaken keypoint placements by nucleus polygon matching on sample 38111. The keypoint placements of cell nuclei are in red boxes. (A) is the cropped patch of the DAPI-stained image; (B) is the cropped patch of the H&E image. (scale bar, 10μm).

1.14. Figure S14

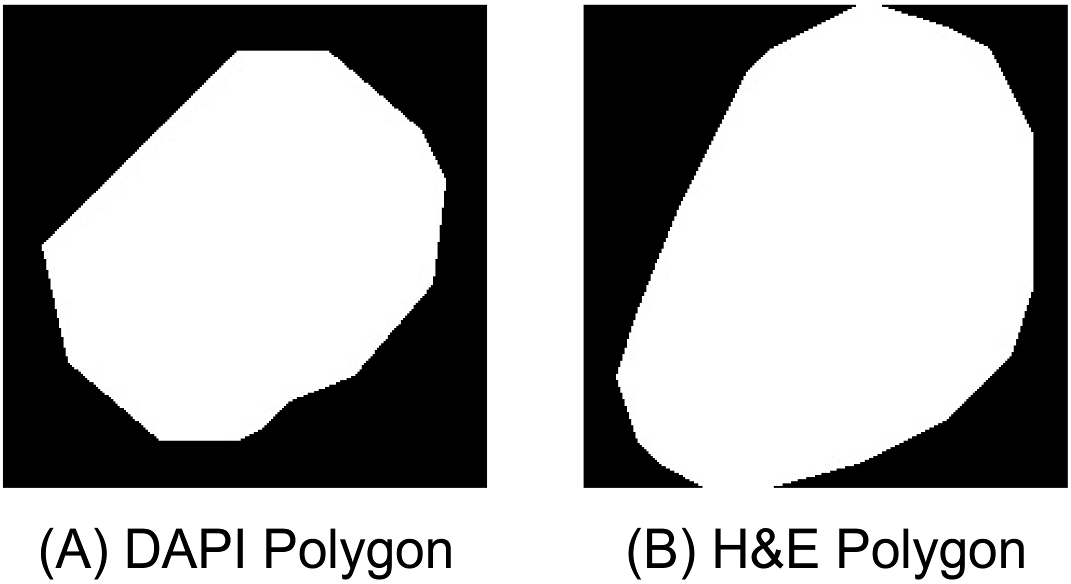

Figure S14. Visualization result of the cell nuclei polygons of two keypoint placements on DAPI-stained image and H&E image in the case of removing the mistaken keypoint placements by nucleus polygon matching on sample 38111. The average overlap index is set as 0.9 and the calculated overlap index between (A) and (B) is 0.847. (A) is the nucleus polygon of the DAPI-stained image; (B) is the nucleus polygon of the H&E image.

1.15. Figure S15

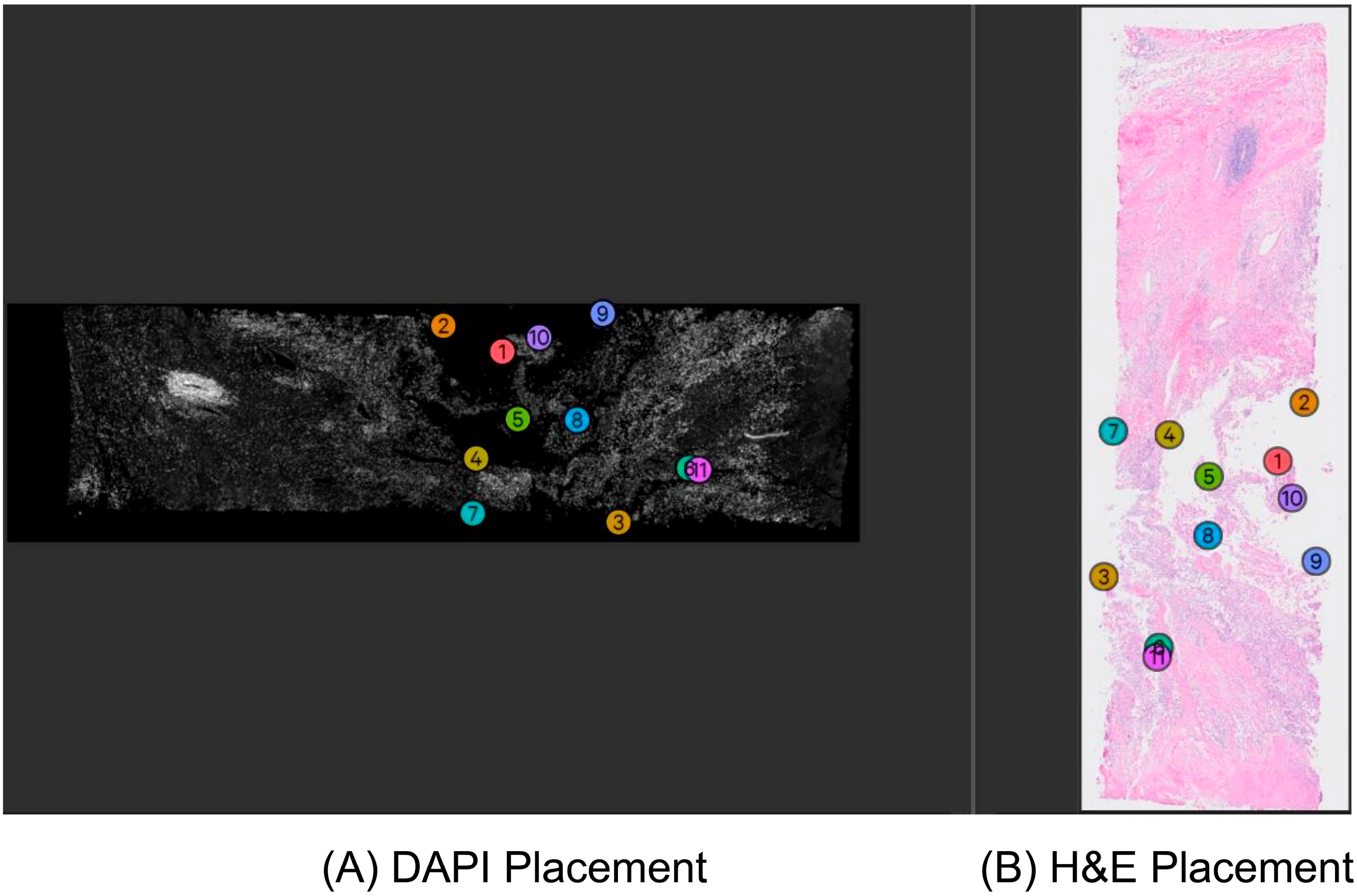

Figure S15. Visualization result of keypoint placements by Xenium-Align in Xenium Explorer software on the sample of cancer kidney. (A) is the keypoint placement of the DAPI-stained image; (B) is the keypoint placement of the H&E image.

2. Supplementary Tables

2.1. Table S1

Table S1. The details about the experimental Xenium dataset with FF preservation method, in which from F59 to 5582 are 12 human kidney samples. N_Cell is the number of cells in each sample, and Condition is the corresponding state of each sample. REF means the reference or healthy kidney samples, CKD means the samples taken from the patients with chronic kidney diseases, and SLE means the samples taken from the patients with autoimmune disease of Lupus.

Sample	F59	20012	26429	36816	3723	3775
N_Cell	26213	40788	52857	49139	102987	363744
Condition	REF	SLE	SLE	SLE	REF	REF
Sample	3781	38111	40440	40610	40775	5582
N_Cell	36678	71700	30865	92438	50394	35352
Condition	REF	SLE	DKD	DKD	DKD	DKD

2.2. Table S2

Table S2. The details about the hyper-parameter settings of nucleus segmentation models on the H&E images of 12 Xenium samples with the FF preservation method.

Sample	F59	20012	26429	36816	3723	3781
Nucleus Segmentation	channel_cellpose=1, flow_threshold=0.8, min_size=15 (Cellpose Model)					
Sample	40440	40610	40775	5582	3775	38111
Nucleus Segmentation	channel_cellpose=1, flow_threshold=0.8, min_size=15 (Cellpose Model)				prob_thresh=0.3 (StarDist Model)	

2.3. Table S3

Table S3. The segmentation results of the nucleus image segmentation model on each sample. N_Segment is the number of segments in the output of image segmentation model and N_Cell is the number of cells in each sample, respectively. Segment_Ratio is the ratio of segment numbers to the total number of cells in each sample.

Sample	F59	20012	26429	36816	3723	3775
N_Segment	18321	28757	33517	32794	73247	252544
N_Cell	26213	40788	52857	49139	102987	363744
Segment_Ratio	0.699	0.705	0.634	0.667	0.711	0.694
Sample	3781	38111	40440	40610	40775	5582
N_Segment	25825	48620	20727	66520	37991	21524
N_Cell	36678	71700	30865	92438	50394	35352
Segment_Ratio	0.704	0.678	0.672	0.720	0.754	0.609

2.4. *Table S4*

Table S4. The details about the keypoints generation on 12 human kidney Xenium samples with the FF preservation method, using a minimum overlap threshold value of 0.92. N_Keypoints is the number of generated keypoints using Xenium-Align. N_Accurate is the number of accurate keypoints in the output of our proposed keypoints identification method. R_Time is the computational time in hours of keypoint identification based on the segmented H&E image and the extracted DAPI-stained image.

Sample	F59	20012	26429	36816	3723	3775
N_Keypoints	20	17	16	16	17	16
N_Accuate	20	17	16	16	17	16
R_Time	14.693	17.893	143.802	20.519	88.551	80.411
Sample	3781	38111	40440	40610	40775	5582
N_Keypoints	16	17	17	20	16	19
N_Accuate	15	17	17	20	16	19
R_Time	69.229	200.817	60.131	25.950	65.303	48.748

2.5. Table S5

Table S5. The details about the hyper-parameter settings of Xenium-Align in the cases of mistaken keypoint placements to show the functional utility of Delaunay triangulation graph and nucleus polygon matchings on two samples of 20012 and 38111.

Sample	Segmentation Method	DAPI Search	Enhanced Assessment	Epoch Num	Case Description
20012	Cellpose	crop_radius_ratio=0.125, extracted_region_min=50	crop_radius_pixel=400, center_move_pixel=300, cell_num_each_epoch=100	1	Delaunay Triangulation Graph Matching
38111	StarDist	crop_radius_ratio=0.06, extracted_region_min=50	crop_radius_pixel=400, center_move_pixel=300, cell_num_each_epoch=100	4	Nucleus Polygon Matching

2.6. Table S6

Table S6. The details about the hyper-parameter settings of Xenium-Align and the results of keypoint generation on two human kidney and one human skin samples with FFPE preservation method. N_Epoch is the number of epochs in conducting Xenium-Align for generating keypoints. N_Keypoints is the number of generated keypoints using Xenium-Align. N_Accurate is the number of accurate keypoints in the output of our proposed keypoints identification method. R_Time is the computational time in hours of keypoint identification based on the segmented H&E image and the extracted DAPI-stained image.

Sample	DAPI Search	Evaluation and Matching	N_Epoch	N_Keypoints	N_Accurate	R_Time
Kidney-Cancer	crop_radius_ratio=0.125, extracted_region_min=50	crop_radius_pixel=200, center_move_pixel=150, cell_num_each_epoch=100, overlap_ave_threshold=0.9, keypoints_min=10	52	11	11	28.364
Non-Diseased - Kidney			3	15	15	2.962
Non-Diseased - Skin	crop_radius_ratio=0.06, extracted_region_min=50		3	13	13	1.445
